# The MdERF17–MdbHLH149 Module Mediates Ethylene‐Induced Starch Degradation Through the Transcriptional Repression of α‐Amylase *MdAMY1* in Apple

**DOI:** 10.1111/pbi.70561

**Published:** 2026-01-23

**Authors:** Fan Xiao, Chu‐Kun Wang, Jiu‐Cheng Zhang, Xin‐Yue Jian, Ying Xiang, Wang‐Jiang Zhang, Jin‐Chao Meng, Wen‐Yan Wang, Da‐Gang Hu

**Affiliations:** ^1^ National Research Center for Apple Engineering and Technology; Shandong Collaborative Innovation Center of Fruit & Vegetable Quality and Efficient Production; College of Horticulture Science and Engineering Shandong Agricultural University Tai'an Shandong China; ^2^ Key Laboratory of Special Fruits and Vegetables Cultivation Physiology and Germplasm Resources Utilization of Xinjiang Production and Construction Corps, Department of Horticulture, College of Agriculture Shihezi University Xinjiang China

**Keywords:** apple, ethylene signalling, starch degradation, sugar metabolism, transcriptional regulation

## Abstract

The ripening of climacteric fruits is characterised by a sharp increase in ethylene production, coinciding with the conversion of starch into soluble sugars. However, the regulatory interplay between ethylene and starch degradation in apple remains largely unclear. Here, we report a negative correlation between starch accumulation and ethylene levels during late fruit development. Integrated transcriptomic analysis identified the α‐amylase gene *MdAMY1* as a key component of the ethylene–starch regulatory pathway. Functional characterisation confirmed that *MdAMY1*, an ethylene‐responsive gene, acts as a positive regulator of starch‐to‐sugar conversion. Biochemical assays showed that the basic helix–loop–helix (bHLH) transcription factor MdbHLH149 directly represses *MdAMY1* transcription. Furthermore, MdERF17—a negative regulator in ethylene signalling—interacts with MdbHLH149 and synergistically enhances this repression. A combination of GUS staining, quantitative enzyme activity assays and VIGS‐based transient transformation demonstrated that the MdERF17–MdbHLH149–*MdAMY1* module acts downstream of ethylene signalling to control starch degradation. Collectively, these findings establish that ethylene facilitates starch degradation by negatively regulating the MdERF17–MdbHLH149–*MdAMY1* repression module.

## 1 Introduction

Starch functions as a key energy reserve in plants, primarily providing metabolic energy and playing an essential role in various physiological processes, including metabolism, growth, development and fruit ripening (Sulpice et al. 2009). In most fleshy fruits, starch undergoes a marked transition during development: it accumulates gradually in early stages but declines rapidly upon maturation, being converted into soluble sugars. This metabolic shift elevates the fruit's sugar content, thereby contributing to its sweetness and flavour profile (Alexander and Grierson 2002; Wang, Wen, et al. 2025).

Starch is a polysaccharide polymer composed of glucose units and is categorised into two main types: amylose and amylopectin (Smith and Zeeman 2020). During fruit ripening, the decline in starch content results from enzymatic degradation, a process that involves the coordinated action of multiple enzymes and a cascade of biochemical reactions such as phosphorylation, dephosphorylation and hydrolysis (Liu et al. 2023). Central to this process are two key starch‐hydrolyzing enzymes, α‐amylase and β‐amylase, which catalyse the cleavage of α‐1,4‐glycosidic bonds in starch, yielding products such as dextrins, oligosaccharides and monosaccharides (Fulton et al. 2008). Consistent with their important roles, starch levels in the *bam3* mutant are significantly higher than in the wild type (Fulton et al. 2008). Further supporting their functional significance, two α‐amylase genes (*AdAMY1* and *AdAMY3*) and four β‐amylase genes (*AdBAM1*, *AdBAM3.1*, *AdBAM3.2* and *AdBAM9*) have been identified, with experimental evidence confirming their involvement in the regulation of starch metabolism during fruit development (Nardozza et al. 2013).

Beyond the enzymes that directly catalyse starch breakdown, multiple transcription factors (TFs) also play essential roles in regulating the starch degradation pathway. For instance, SlHY5 directly binds to the promoters of starch‐degradation genes *SlBAM1*, *SlBAM3* and *SlBAM8* to activate their transcription (Dong et al. 2021). Similarly, MabZIP21 promotes starch breakdown by upregulating the expression of *MaBAM4*, *MaBAM7* and *MaAMY3* (Xu et al. 2024). The ERF transcription factor MaERF95L accelerates starch degradation and sugar accumulation during banana ripening by activating the expression of six genes related to starch degradation and sucrose synthesis (Xie et al. 2025), while MabHLH6 acts as a transcriptional activator by binding to E‐box motifs in the promoters of 11 starch degradation‐related genes (Xiao et al. 2018). Additionally, MdAREB2 facilitates starch degradation by transcriptionally activating several amylase genes (Ma et al. 2017), and MdWRKY32 participates in starch metabolism through specific binding to the *MdBam5* promoter (Li et al. 2021).

Ethylene, a phytohormone ubiquitous in higher plants, plays a pivotal role in regulating essential physiological processes such as seed germination, fruit ripening and leaf senescence (Ju et al. 2015; Wang et al. 2021; Li et al. 2023; Varshney et al. 2023; Xu et al. 2023). Its biosynthesis begins with S‐adenosylmethionine and proceeds through a two‐step enzymatic pathway catalysed sequentially by 1‐aminocyclopropane‐1‐carboxylate synthase (ACS) and 1‐aminocyclopropane‐1‐carboxylate oxidase (ACO) (Thomas 2022). Ethylene signalling is transduced via a conserved pathway involving central components such as ETHYLENE INSENSITIVE 2 (EIN2), EIN3/EIN3‐like proteins (EILs) and ethylene response factors (ERFs). Upon ethylene perception, these regulators orchestrate the transcriptional reprogramming of downstream target genes (Hua et al. 1998; Yang et al. 2015). For example, AdEIL2 and AdEIL3 promote ripening by activating ripening‐associated genes in kiwi fruit (Yin et al. 2010). Similarly, SlEIL proteins directly upregulate key ripening regulators such as *NOR*, *RIN* and *FRUITFULL1* in response to ethylene (Huang et al. 2022). In apple, ethylene modulates fruit quality through multiple mechanisms: it suppresses malate accumulation via the MdERF72‐MdWRKY31 module, which represses *MdALMT9* expression (Wang et al. 2023) and enhances anthocyanin biosynthesis through MdEIL1, which directly activates the promoter of *MdMYB1* to promote fruit pigmentation (An et al. 2018).

The ripening of climacteric fruits involves a well‐defined surge in ethylene production alongside the conversion of starch to sugars. Yet, the specific regulatory interplay between ethylene and the starch degradation pathway in apple is still poorly defined. In this study, we initially identified an α‐amylase gene, *MdAMY1*, which participates in starch degradation during apple fruit development. We further discovered that the transcription factor MdbHLH149 directly suppresses *MdAMY1* expression. Moreover, this repression is potentiated by the interaction between MdERF17 and MdbHLH149, forming a regulatory module that operates downstream of ethylene signalling to fine‐tune starch breakdown.

## 2 Results

### 2.1 *MdAMY1* Participates in Ethylene‐Mediated Starch Degradation

**FIGURE 1:**
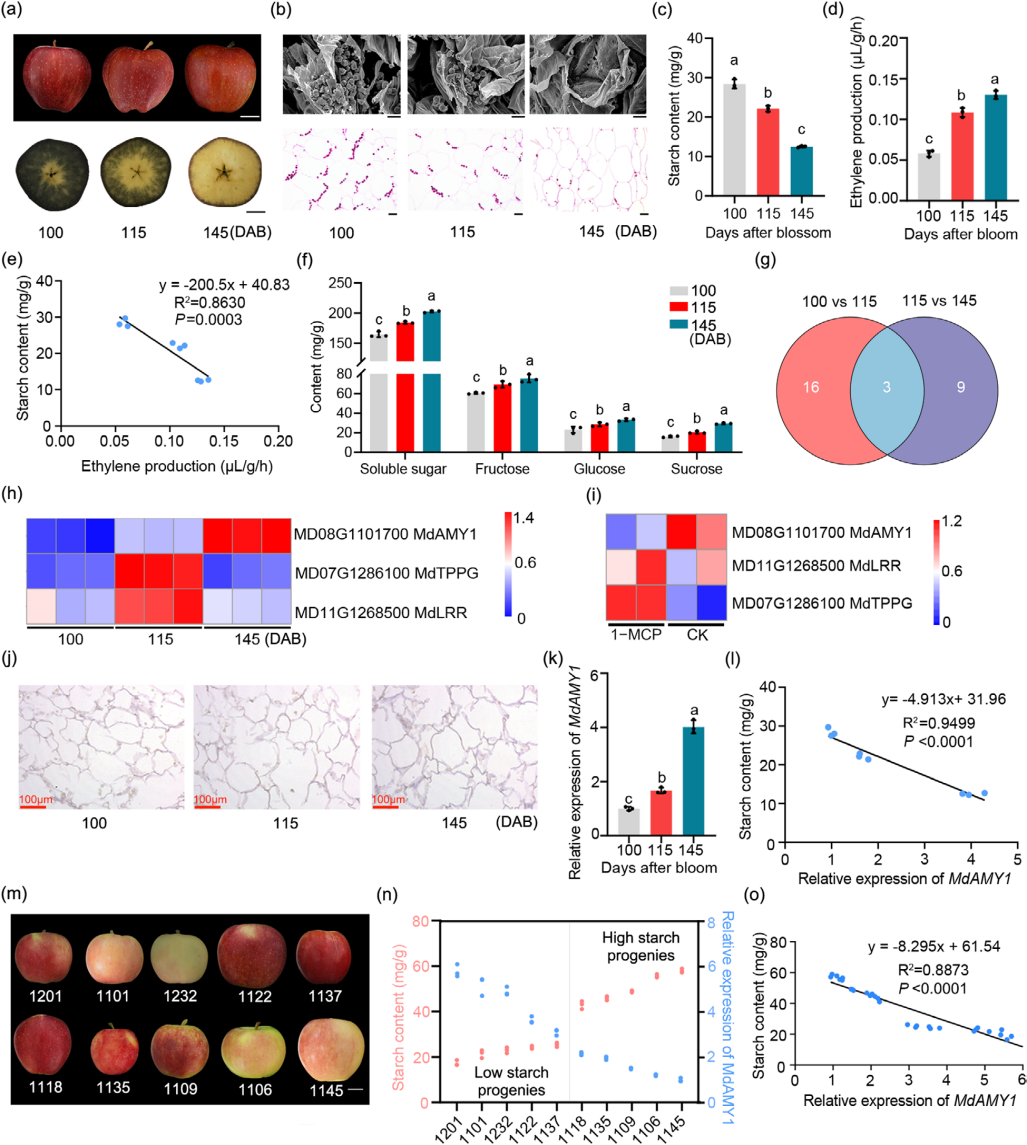
*MdAMY1* is involved in starch degradation and soluble sugar accumulation during apple fruit ripening. (a) Phenotype and KI‐I_2_ staining of ‘Starking Delicious’ apples at 100, 115 and 145 days after bloom (DAB). Scale bar: 2 cm. (b) Scanning electron microscopy (scale bar: 20 μm) and schiff reagent staining (scale bar: 50 μm) of semi‐thin sections showing cell wall and starch granule morphology. (c, d) Quantification of starch content (c) and ethylene emission rate (d) during development. (e) Correlation between starch content and ethylene release rate. (f) Soluble sugar (fructose, sucrose, glucose) contents. Data are mean ± SD (*n* ≥ 3). Different letters indicate significant differences (*p* < 0.05, one‐way ANOVA). (g) Venn diagram of genes enriched in starch and sucrose metabolism pathways (KEGG). (h) Heatmap of starch/sucrose metabolism‐related gene expression at three developmental stages. (i) Expression patterns of 1‐MCP‐treated fruits. (j) RNA in situ hybridization of *MdAMY1* during fruit development. Scale bar: 100 μm. (k) *MdAMY1* expression levels across developmental stages. Data are mean ± SD (*n* ≥ 3). Different letters indicate significant differences (*p* < 0.05, one‐way ANOVA). (l) Correlation between *MdAMY1* expression and starch content during development. (m) Phenotypes of 10 representative F_1_ progeny from ‘Gala’ × ‘Mato 1’ crosses. Scale bar: 2 cm. (n) Starch content and *MdAMY1* expression levels in panel (m) fruits. (o) Correlation between starch content and *MdAMY1* expression in the F_1_ population.

To characterise the dynamic changes in starch content during fruit ripening, ‘Starking Delicious’ apple fruits were sampled at 100, 115 and 145 days after bloom (DAB). Iodine–potassium iodide (I_2_–KI) staining revealed a progressive decrease in staining intensity during maturation, indicating a continuous decline in starch content (Figure 1a). Consistent with this observation, histological analyses via scanning electron microscopy (SEM) and semithin sections stained with schiff reagent showed a gradual reduction in both the number and size of starch granules over the same period (Figure 1b). We further analysed the relationship between starch content and ethylene production during ripening. Starch levels decreased markedly, whereas ethylene emission increased significantly (Figure 1c,d), and a strong negative correlation was observed between the two parameters (Figure 1e). Paradoxically, despite the ongoing starch degradation, the content of soluble sugars (fructose, glucose, sucrose) showed a significant reduction at this stage (Figure 1f).

To assess the regulatory role of ethylene in starch metabolism, apple fruits were treated with the ethylene precursor 1‐aminocyclopropane‐1‐carboxylic acid (ACC) and the ethylene action inhibitor 1‐methylcyclopropene (1‐MCP). I_2_–KI staining indicated that ACC treatment accelerated starch degradation, yielding a smaller staining area compared to the control, while 1‐MCP treatment retained more starch, as reflected by a larger staining area (Figure S1a). Quantitative analysis confirmed that ACC treatment significantly decreased starch content and promoted ethylene release, whereas 1‐MCP treatment maintained higher starch levels and suppressed ethylene production (Figure S1b,c). Again, the starch content was negatively correlated with ethylene production (Figure S1d). In line with these changes, the levels of soluble sugars, including fructose, glucose and sucrose, were increased in ACC‐treated fruits and decreased in 1‐MCP‐treated fruits relative to the control (Figure S1e).

To identify the key genes involved in ethylene‐mediated starch degradation during fruit maturation, transcriptomic analysis was performed on ‘Starking Delicious’ apple fruit collected at 100, 115 and 145 DAB. Comparative transcriptome profiling identified 495 upregulated and 527 downregulated genes at 115 DAB compared to 100 DAB (Figure S2a), and 276 upregulated and 463 downregulated genes at 115 DAB relative to 145 DAB (Figure S2b). Kyoto encyclopedia of genes and genomes (KEGG) pathway analysis of these differentially expressed genes showed significant enrichment in metabolic pathways, biosynthesis of secondary metabolites, and starch and sucrose metabolism (Figure S2c,d). Gene ontology (GO) analysis further indicated substantial changes in biological processes, cellular components and molecular functions (Figure S2e,f). From the starch and sucrose metabolism pathway, we identified three consistently enriched genes—*MdAMY1*, *MdTPPG* and *MdLRR*—across both comparative groups (Figure 1g; Data S1 and S2). Temporal expression analysis during fruit ripening revealed that only *MdAMY1* exhibited an expression pattern inversely correlated with starch content and closely aligned with the progression of starch degradation (Figure 1h). Consistent with ethylene regulation, *MdAMY1* expression was suppressed (Figure 1i; Data S3) in 1‐MCP‐treated apples based on a published transcriptome dataset (Wang et al. 2023). RNA in situ hybridization and quantitative PCR with reverse transcription (RT–qPCR) confirmed that *MdAMY1* expression progressively increased during ripening (Figure 1j,k; Figure S3), and its expression was negatively correlated with starch content (Figure 1l).

To further validate this relationship, we analysed progenies from a ‘Gala’ × ‘Mato 1’ F_1_ hybrid population segregated for starch content (Figure 1m). *MdAMY1* expression was significantly downregulated in high‐starch progeny and upregulated in low‐starch individuals, and again negatively correlated with starch content (Figure 1n,o).

Collectively, these results demonstrate that *MdAMY1* acts as a key regulator of ethylene‐mediated starch degradation during apple fruit ripening.

### 2.2 MdAMY1 Functions as a Cell Wall‐Localised α‐Amylase That Promotes Starch Degradation and Soluble Sugar Accumulation in Apple

**FIGURE 2:**
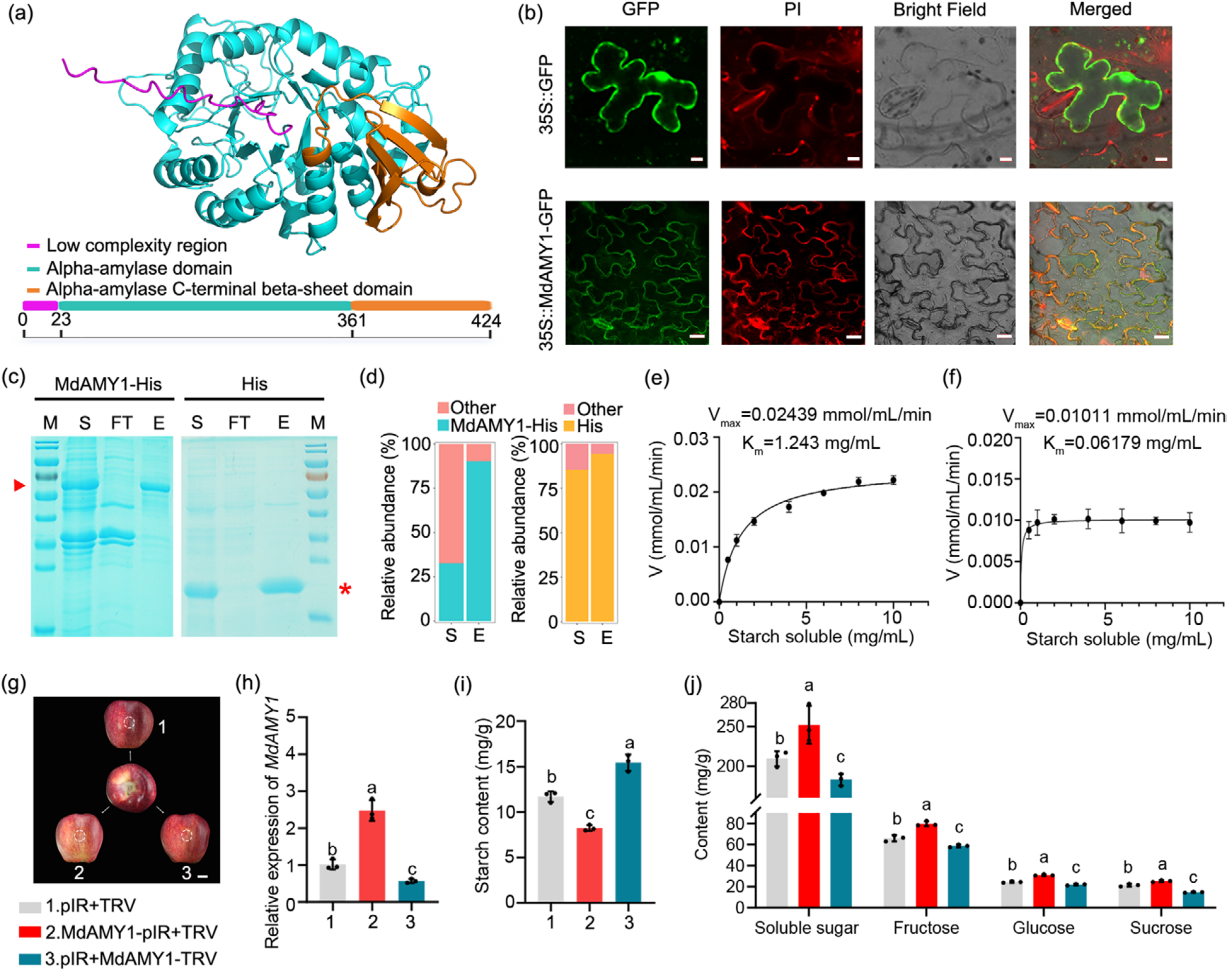
*MdAMY1* promotes starch degradation and soluble sugar accumulation in apple. (a) Domain architecture of the MdAMY1 protein. (b) Subcellular localization of 35S::MdAMY1‐GFP transiently expressed in *Nicotiana benthamiana* leaves. Scale bar: 20 μm. (c) SDS‐PAGE analysis of purified recombinant proteins. Supernatant (S), flow‐through (FT) and elution fractions (E; 200 mM imidazole) were stained with Coomassie Blue. Red triangles indicate target proteins. The empty pET32a vector produced a ~20.5 kDa His‐tagged fusion, while recombinant MdAMY1 migrated at ~66.7 kDa. (d) Densitometric quantification of protein bands using ImageJ (v1.53). (e, f) Enzyme kinetics of purified recombinant MdAMY1‐His (e) and His‐tag control (f). Data are mean ± SD of three biological replicates. (g) Phenotype of ‘Starking Delicious’ apples after infiltration with overexpression (pIR, MdAMY1‐pIR) or silencing (TRV, MdAMY1‐TRV) vectors. Scale bar: 2 cm. (h) RT‐qPCR analysis of *MdAMY1* expression in infiltrated regions from (g). (i, j) Metabolic analysis of injection sites: Starch (i) and soluble sugars (fructose, sucrose, glucose) (j). Data are mean ± SD (*n* ≥ 3 biological replicates, two apples each). Different letters indicate significant differences (*p* < 0.05, one‐way ANOVA).

To investigate the function of MdAMY1, we first analysed its protein domain architecture and identified a low‐complexity region, an α‐amylase domain, and a C‐terminal β‐sheet domain characteristic of α‐amylases (Figure 2a). To determine its subcellular localization, a 35S::MdAMY1‐GFP fusion construct was transiently expressed in tobacco leaves. Confocal microscopy showed that the GFP signal of MdAMY1 colocalized with the red fluorescence of propidium iodide (PI, a cell wall marker), indicating that MdAMY1 is localised to the cell wall (Figure 2b). We next assessed the enzymatic activity of MdAMY1 in vitro. Recombinant MdAMY1‐His protein was purified using Ni‐NTA affinity chromatography. SDS‐PAGE analysis confirmed efficient removal of impurities in the flow‐through (FT) and successful recovery of purified MdAMY1‐His protein in the elution fraction (E) (Figure 2c,d). Incubation of the purified MdAMY1‐His protein (E) with soluble starch resulted in significant starch degradation, whereas neither the His‐tagged control protein nor the flow‐through fraction exhibited such activity (Figure 2e,f; Figure S4a).

To further investigate the physiological role of *MdAMY1* in starch degradation in vivo, we performed virus‐mediated transient transformation in ‘Starking Delicious’ apple fruit. Fruits were infiltrated with the following constructs: pIR + TRV (empty vector control), MdAMY1‐pIR + TRV (overexpression of *MdAMY1*), and pIR + MdAMY1‐TRV (silencing of *MdAMY1*) (Figure 2g). *MdAMY1* overexpression significantly reduced starch content and concurrently increased the levels of the measured soluble sugars (Figure 2h–j). In contrast, *MdAMY1* silencing led to a marked increase in starch content and a concomitant decrease in these soluble sugars (Figure 2h–j).

To further corroborate these findings, we generated two independent, stable *MdAMY1‐*overexpressing transgenic calli, designated MdAMY1‐OE2 and MdAMY1‐OE4 (Figure S4b,c). Consistent with the transient expression results, both overexpression lines displayed significantly reduced starch content and elevated levels of soluble sugars, fructose, glucose and sucrose compared to the wild‐type control (Figure S4d,e).

Collectively, these results demonstrate that *MdAMY1* positively regulates starch degradation and facilitates the conversion of starch into soluble sugars in apple.

### 2.3 MdbHLH149 Interacts With the *MdAMY1* Promoter and Functions as a Negative Regulator of Starch‐To‐Sugar Conversion

**FIGURE 3:**
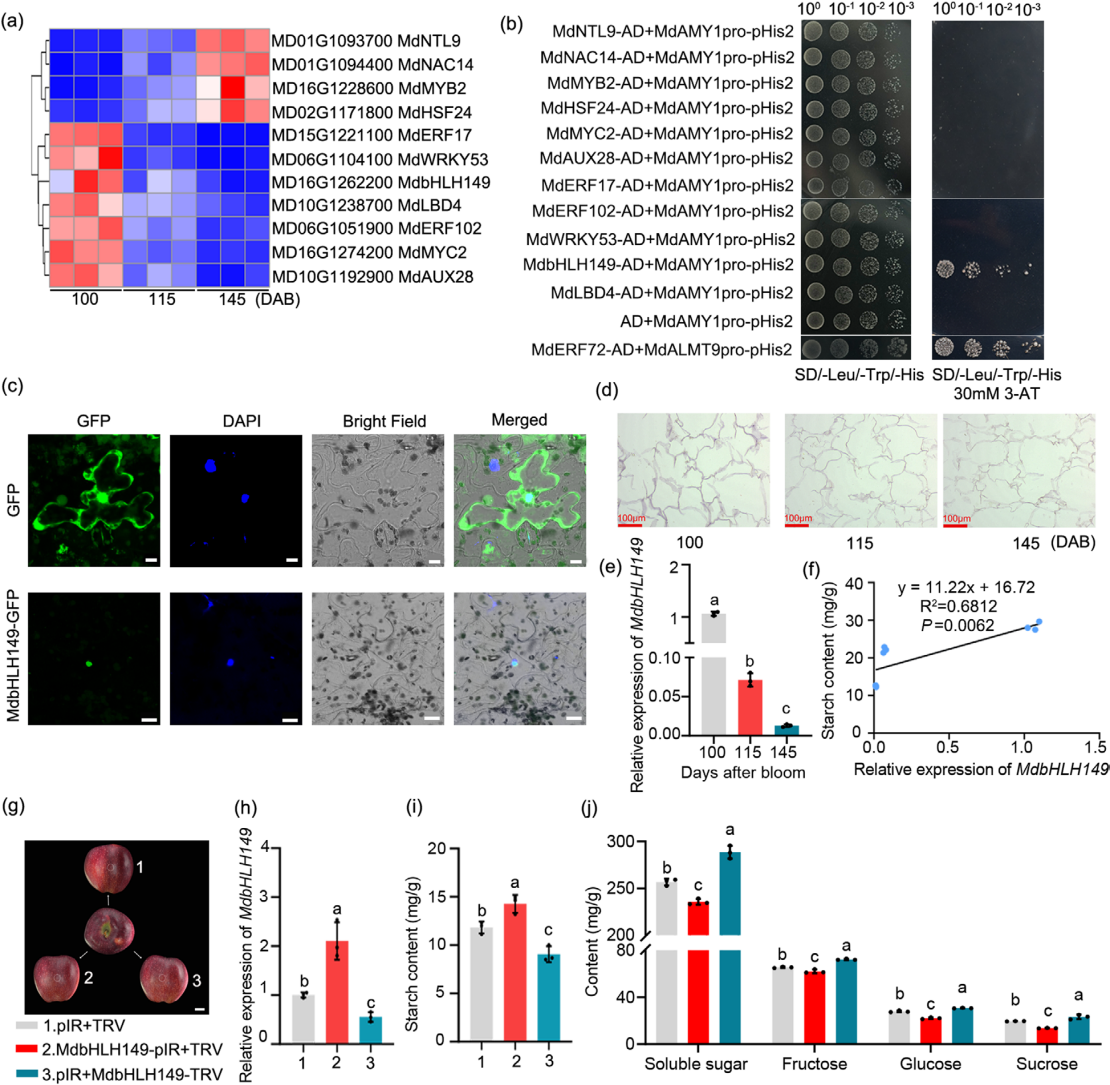
MdbHLH149 interacts with *MdAMY1* promoter and represses starch‐to‐sugar conversion. (a) Transcriptional dynamics of differentially expressed transcription factors (TFs) in apple fruit at 100, 115 and 145 DAB. (b) Yeast one‐hybrid assay showing MdbHLH149 binding to the *MdAMY1* promoter. Yeast cells co‐transformed with pGAD424‐MdbHLH149 and MdAMY1pro‐pHis2 were grown on SD/–Leu/–Trp/–His medium containing 30 mM 3‐amino‐1,2,4‐triazole (3‐AT) for 3 days at 28°C. The empty pGAD424 vector served as a negative control, and the MdERF72‐AD+MdALMT9pro‐pHis2 was used as the positive control. (c) Subcellular localization of 35S::MdbHLH149‐GFP in tobacco epidermal cells. Scale bar: 20 μm. (d) RNA in situ hybridization of MdbHLH149 in apple fruit tissues at 100, 115 and 145 DAB. Scale bar: 100 μm. (e) RT‐qPCR analysis of *MdbHLH149* expression during fruit development (100–145 DAB). (f) Correlation between *MdbHLH149* transcript levels and starch content across developmental stages. (g) Phenotype of ‘Starking Delicious’ apples after transient transformation with overexpression (pIR, MdbHLH149‐pIR) or silencing (TRV, MdbHLH149‐TRV) vectors. Scale bar: 2 cm. (h) RT‐qPCR analysis of *MdbHLH149* expression in infiltrated regions from (g). (i, j) Metabolic profiles of infiltrated zones: Starch (i) and soluble sugars (fructose, sucrose, glucose) (j). Data are mean ± SD (*n* ≥ 3 biological replicates, two apples each). Different letters indicate significant differences (*p* < 0.05, one‐way ANOVA).

To elucidate the molecular mechanism by which *MdAMY1* regulates starch degradation, we analysed differentially expressed transcription factors using transcriptome data from fruit samples of ‘Starking Delicious’ apples collected at 100, 115 and 145 DAB. Expression profiling revealed that transcript levels of *MdNTL9*, *MdNAC14*, *MdMYB2* and *MdHSF24* increased significantly during fruit ripening, whereas those of *MdERF17*, *MdWRKY53*, *MdbHLH149*, *MdLBD4*, *MdERF102*, *MdMYC2* and *MdAUX28* decreased significantly (Figure 3a; Data S4). We then examined the binding of these transcription factors to the *MdAMY1* promoter using a yeast one‐hybrid (Y1H) assay. The *MdAMY1* promoter was cloned into the pHis2 vector, and the coding sequences of the selected transcription factors were fused to the activation domain in the pGAD424 vector. Among them, MdbHLH149 activated the reporter gene expression driven by the *MdAMY1* promoter, as evidenced by enhanced yeast growth on selective medium compared to the control (Figure 3b).

To determine the subcellular localization of MdbHLH149, a 35S::MdbHLH149‐GFP fusion construct was transiently expressed in tobacco leaves. Confocal microscopy revealed that the GFP signal colocalized with 4′,6‐diamidino‐2‐phenylindole (DAPI) staining, confirming the nuclear localization of MdbHLH149 (Figure 3c). Furthermore, RNA in situ hybridization and RT‐qPCR analyses demonstrated that *MdbHLH149* expression was significantly downregulated during fruit maturation (Figure 3d,e; Figure S5a). Notably, *MdbHLH149* transcript levels were positively correlated with starch content (Figure 3f).

To investigate the role of *MdbHLH149* in starch degradation, we performed transient overexpression and silencing of *MdbHLH149* in ‘Starking Delicious’ apple fruits using a virus‐induced gene silencing (VIGS) system with the following constructs: pIR + TRV (empty vector control), MdbHLH149‐pIR + TRV (overexpression of *MdbHLH149*), and pIR + MdbHLH149‐TRV (silencing of *MdbHLH149*) (Figure 3g). Overexpression of *MdbHLH149* led to a significant increase in starch content (Figure 3h,i) and a decrease in soluble sugars, fructose, glucose and sucrose (Figure 3j). Conversely, silencing of *MdbHLH149* significantly reduced starch content and increased the levels of soluble sugars, fructose, glucose and sucrose (Figure 3h–j). Consistent with these findings, the relative expression of *MdbHLH149* and starch content in the two independent *MdbHLH149*‐overexpressing calli lines (MdbHLH149‐OE5 and MdbHLH149‐OE8) of ‘Orin’ apple was significantly higher than that of the wild type, while the soluble sugar, fructose, glucose and sucrose levels were reduced compared to those in the wild type (Figure S5b–e). Collectively, these results demonstrate that MdbHLH149 acts as a negative regulator of starch‐to‐soluble sugar conversion during apple fruit ripening.

### 2.4 MdbHLH149 Represses *MdAMY1* Expression to Suppress Starch Degradation

**FIGURE 4:**
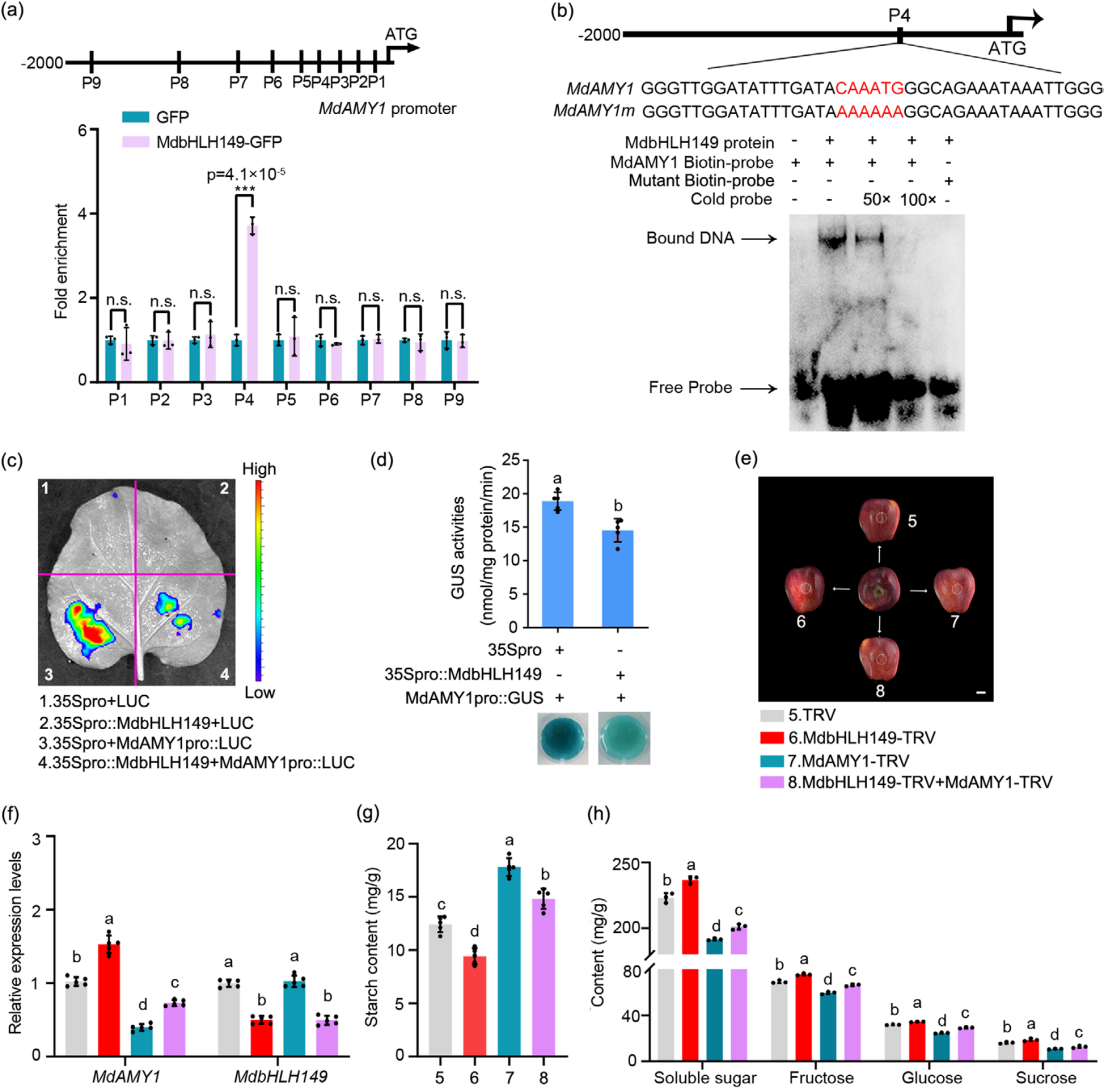
MdbHLH149 represses starch degradation by transcriptionally inhibiting *MdAMY1* expression. (a) Chromatin immunoprecipitation (ChIP) assays demonstrating direct binding of MdbHLH149 to the P4 region of the *MdAMY1* promoter. Black bars represent amplified genomic regions. Data are mean ± SD of three independent experiments. ***p < 0.001 versus control (two‐tailed Student's *t*‐test). (b) Electrophoretic mobility shift assay (EMSA) showing specific binding of MdbHLH149 to a *cis*‐element in the *MdAMY1* promoter. Biotin‐labelled probes were used with unlabeled competitor; a probe with mutated core motif (5′‐CAAATG‐3′ to 5′‐AAAAAA‐3′) abolished binding. Protein‐free sample served as negative control. (c) Luciferase imaging of tobacco leaves 2.5 days after Agrobacterium infiltration with effector (35S::MdbHLH149 in pGreenII62‐SK) and reporter (MdAMY1pro::Luc in pGreenII0800‐Luc) constructs. (d) GUS activity assays in apple calli co‐transformed with MdAMY1pro::GUS and 35S::MdbHLH149. Data are mean ± SD (*n* ≥ 3). Different letters indicate significant differences (*p* < 0.05, Student's *t*‐test). (e) Phenotype of ‘Starking Delicious’ apples 7 days after infiltration with indicated VIGS constructs. Scale bar: 2 cm. (f) RT‐qPCR analysis of *MdAMY1* and *MdbHLH149* expression in infiltrated fruit regions from (e). (g, h) Metabolic analysis of injection sites: Starch (g) and soluble sugars (fructose, sucrose, glucose) (h). Data are mean ± SD (*n* ≥ 3 biological replicates, two apples each). Different letters indicate significant differences (*p* < 0.05, one‐way ANOVA).

To further dissect the transcriptional regulatory mechanism between MdbHLH149 and *MdAMY1*, we conducted a series of molecular experiments. Chromatin immunoprecipitation (ChIP‐PCR) assays using MdbHLH149‐GFP calli revealed significant enrichment of the E‐box‐containing promoter region of *MdAMY1* (P4) compared to the GFP control (Figure 4a). Electrophoretic mobility shift assays (EMSA) further confirmed that MdbHLH149 directly binds to a DNA probe containing the E‐box cis‐element within the *MdAMY1* promoter (Figure 4b). To assess the functional consequence of this binding, transient transcriptional activity assays were performed in tobacco leaves using 35S::MdbHLH149 as the effector and MdAMY1pro::LUC as the reporter (Figure S6a). These assays showed that MdbHLH149 significantly suppressed the LUC activity driven by the *MdAMY1* promoter (Figure 4c). Consistent with this result, GUS staining and quantitative assays demonstrated markedly reduced GUS activity in transgenic calli co‐expressing MdAMY1pro::GUS and 35S::MdbHLH149 compared to controls containing MdAMY1pro::GUS and the empty 35S promoter vector (Figure 4d and Figure S6b). Collectively, these findings demonstrate that MdbHLH149 directly binds to the *MdAMY1* promoter and functions as a transcriptional repressor of *MdAMY1* expression.

To determine whether MdbHLH149 regulates starch degradation in an *MdAMY1*‐dependent manner, we employed a VIGS system to modulate the expression of *MdbHLH149* and *MdAMY1* in ‘Starking Delicious’ apple fruits. Fruits were infiltrated with the following constructs: TRV (control), MdbHLH149‐TRV, MdAMY1‐TRV, and a combination of MdbHLH149‐TRV + MdAMY1‐TRV (Figure 4e). Silencing of *MdbHLH149* led to a significant downregulation of its own transcript, accompanied by a marked upregulation of *MdAMY1* expression. This resulted in a substantial reduction in starch content and a concurrent increase in soluble sugars, including fructose, sucrose and glucose (Figure 4f–h). In contrast, silencing *MdAMY1* specifically reduced its own expression and strongly suppressed starch degradation, as evidenced by significantly elevated starch levels and pronounced decreases in all measured soluble sugars (Figure 4f–h). In the co‐silencing treatment, the relative expression level of *MdbHLH149* remained low, comparable to the *MdbHLH149* single‐silenced group. Notably, *MdAMY1* expression in the co‐silenced fruits was reduced relative to the control but was higher than in the *MdAMY1* single‐silenced treatment. This intermediate molecular phenotype was reflected in the metabolic data: starch content was higher than in the control but lower than in the *MdAMY1*‐silenced fruits, while soluble sugar levels were correspondingly intermediate between these two groups (Figure 4f–h). Collectively, these results demonstrate that MdbHLH149 acts upstream of *MdAMY1* to repress its expression, thereby inhibiting starch degradation and the subsequent accumulation of soluble sugars.

### 2.5 MdERF17 Potentiates MdbHLH149‐Mediated Transcriptional Repression for *MdAMY1* to Suppress Starch Degradation

**FIGURE 5:**
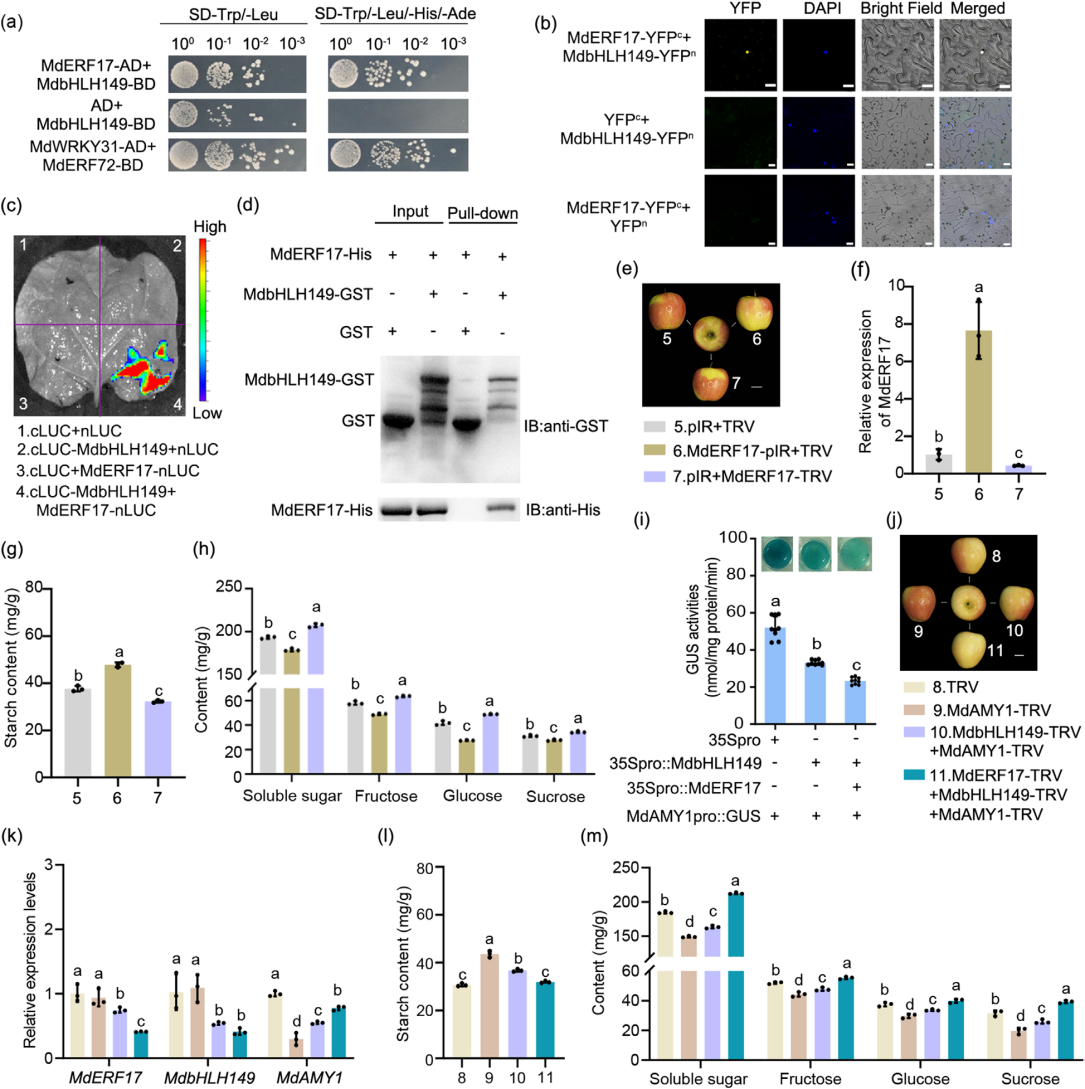
MdERF17 interacts with MdbHLH149 and enhances its transcriptional repression of *MdAMY1*. (a) Yeast two‐hybrid assay showing the interaction between MdERF17 and MdbHLH149. The full‐length coding sequences were cloned into pGAD424 and pGBT9, respectively. AD empty vector and MdbHLH149‐BD were used as negative controls, while MdWRKY31‐AD+MdERF72‐BD was used as the positive control. (b) BiFC assay in tobacco epidermal cells. Co‐expression of MdERF17‐YFP^c^ and MdbHLH149‐YFP^n^ reconstituted YFP fluorescence in the nucleus. Scale bar: 20 μm. (c) Luciferase complementation imaging assay in *Nicotiana benthamiana* leaves infiltrated with cLUC‐MdbHLH149 and MdERF17‐nLUC constructs. Images were taken 3 days after infiltration. (d) Pull‐down assay demonstrating direct interaction between recombinant MdERF17‐His and MdbHLH149‐GST proteins. GST alone served as a negative control. (e) Phenotype of ‘Luli’ apple fruits after transient transformation with overexpression (pIR, MdERF17‐pIR) or silencing (TRV, MdERF17‐TRV) vectors. Scale bar: 2 cm. (f) RT‐qPCR analysis of *MdERF17* expression in infiltrated fruit regions shown in (e). (g, h) Metabolic profiles of infiltrated zones: Starch (g) and soluble sugar (fructose, sucrose, glucose) contents (h). Data are mean ± SD (*n* ≥ 3 biological replicates, two apples each). Different letters indicate significant differences (*p* < 0.05, one‐way ANOVA). (i) GUS activity assays in apple calli co‐transformed with MdAMY1pro::GUS and effector constructs (35S::MdERF17, 35S::MdbHLH149). Activity was measured quantitatively and by histochemical staining. Values are mean ± SD of ≥ 3 replicates. Different letters indicate significant differences (*p* < 0.05, Student's *t*‐test). (j) Phenotype of ‘Luli’ apples 7 days after infiltration with the indicated VIGS vectors. Scale bar: 2 cm. (k) RT‐qPCR analysis of *MdERF17*, *MdbHLH149* and *MdAMY1* expression in infiltrated areas from (j). (l, m) Metabolic analysis of injection sites: Starch (l) and soluble sugar (fructose, sucrose, glucose) contents (m). Data are mean ± SD (*n* ≥ 3 biological replicates, two apples each). Different letters indicate significant differences (*p* < 0.05, one‐way ANOVA).

To identify upstream regulators of MdbHLH149, a yeast two‐hybrid (Y2H) screen was performed using MdbHLH149 as bait against transcription factors differentially expressed during fruit maturation. This screen identified a physical interaction between MdERF17 and MdbHLH149 (Figure 5a). The interaction was further confirmed in planta using bimolecular fluorescence complementation (BiFC) and luciferase (LUC) complementation assays in *Nicotiana benthamiana* leaves. Co‐expression of MdERF17‐YFP^c^ and MdbHLH149‐YFP^n^ reconstituted YFP fluorescence specifically in the nucleus, while control combinations showed no signal (Figure 5b). Similarly, strong LUC activity was detected only in leaves co‐expressing cLUC‐MdbHLH149 and MdERF17‐nLUC (Figure 5c). An in vitro pull‐down assay further validated this interaction: MdbHLH149‐GST, but not GST alone, pulled down MdERF17‐His (Figure 5d). Together, these results demonstrate that MdERF17 interacts with MdbHLH149 both in vivo and in vitro.

To assess the functional role of *MdERF17* in starch metabolism, we transiently overexpressed or silenced *MdERF17* in ‘Luli’ apple fruits using a TRV‐based VIGS system with the following constructs: pIR + TRV (empty vector control), MdERF17‐pIR + TRV (overexpression of *MdERF17*), and pIR + MdERF17‐TRV (silencing of *MdERF17*) (Figure 5e). RT‐qPCR confirmed successful manipulation of *MdERF17* expression at the infiltration sites (Figure 5f). Overexpression of *MdERF17* significantly increased starch accumulation and reduced the levels of soluble sugars, including fructose, glucose and sucrose (Figure 5g,h), a result consistent with observations in *MdERF17*‐overexpressing calli (MdERF17‐OE1 and MdERF17‐OE2), while the expression level of *MdAMY1* also decreased (Figure S7a–d). Conversely, silencing *MdERF17* decreased starch content and increased soluble sugar levels (Figure 5g,h), indicating that *MdERF17* suppresses starch degradation.

We then investigated whether the MdERF17–MdbHLH149 interaction influences the transcriptional repression of *MdAMY1*. GUS activity assays in calli showed that co‐expression of *MdERF17* and *MdbHLH149* resulted in stronger repression of the *MdAMY1* promoter than expression of *MdbHLH149* alone (Figure 5i), suggesting that MdERF17 enhances MdbHLH149‐mediated transcriptional repression. To define the functional hierarchy of the MdERF17‐MdbHLH149‐*MdAMY1* module in starch degradation, we conducted combinatorial silencing experiments using the VIGS system (Figure 5j). Simultaneous silencing of *MdAMY1* and *MdbHLH149* led to higher *MdAMY1* expression, lower starch content, and higher soluble sugar levels compared with silencing *MdAMY1* alone (Figure 5k–m). Furthermore, triple silencing of *MdERF17*, *MdbHLH149* and *MdAMY1* resulted in even higher *MdAMY1* transcript levels, a further reduction in starch, and a more pronounced increase in soluble sugars compared to co‐silencing *MdbHLH149* and *MdAMY1* (Figure 5k–m).

In the hybrid population, *MdERF17* and *MdbHLH149* showed higher expression in low‐starch progenies and lower expression in high‐starch progenies. Their transcript abundance exhibited a negative correlation with starch level (Figure S8a–c).

In summary, these findings demonstrate that MdERF17 interacts with MdbHLH149 and potentiates its transcriptional repression of *MdAMY1*, thereby collectively suppressing starch degradation and contributing to natural variation in starch content within the hybrid population.

### 2.6 Ethylene Signalling Integrates With the MdERF17–MdbHLH149– *MdAMY1* Module to Regulate Starch Degradation

**FIGURE 6:**
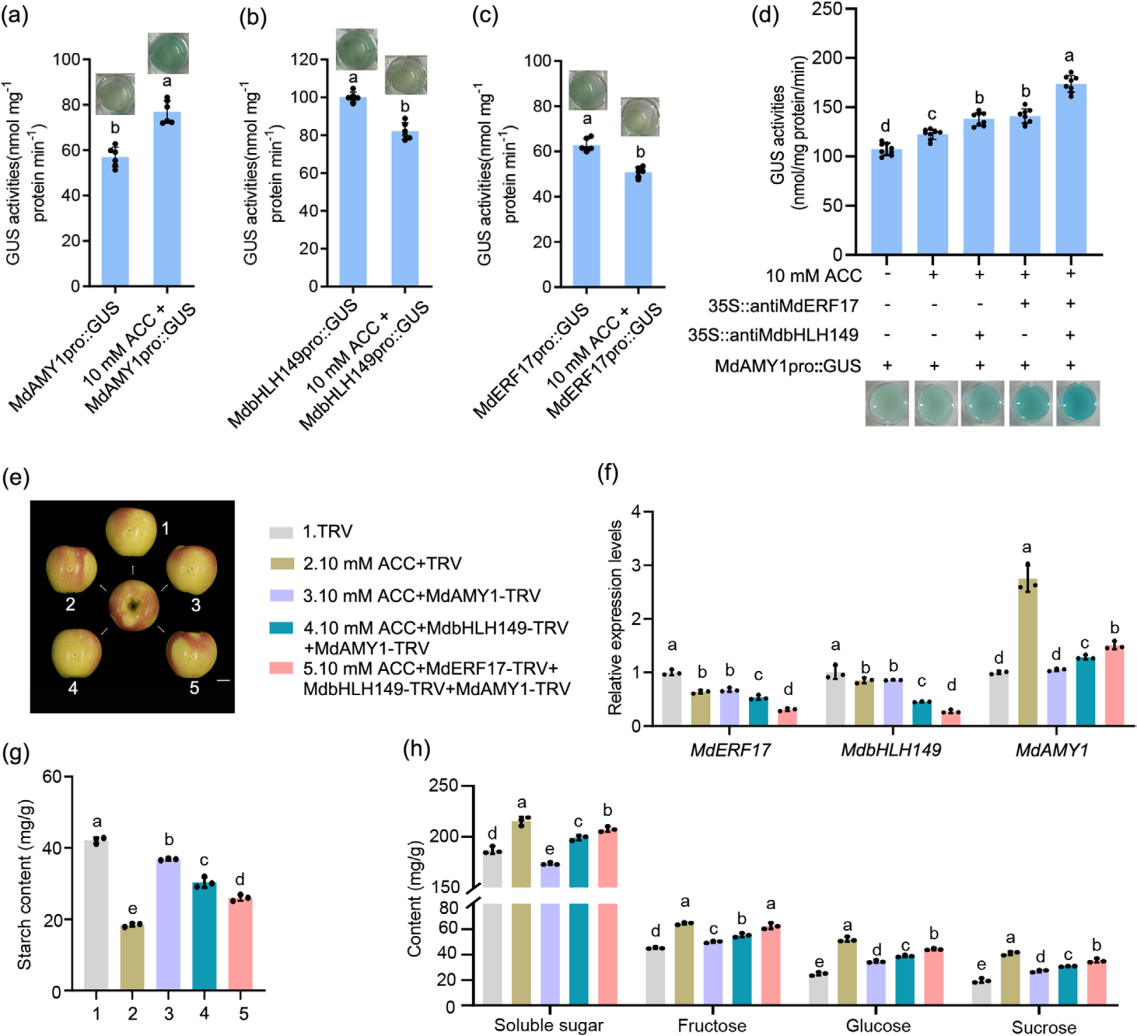
Ethylene regulates starch degradation via the MdERF17‐MdbHLH149‐MdAMY1 module in apple fruit. (a–c) GUS activity assays in apple calli transformed with MdAMY1pro::GUS (a)/MdbHLH149pro::GUS (b)/MdERF17pro::GUS (c), treated with 10 mM ACC or untreated. GUS activity was quantified enzymatically and visualised by histochemical staining. Data represent mean ± SD of at least three biological replicates. Different letters indicate significant differences (*p* < 0.05, Student's *t*‐test). (d) GUS activity assays in apple calli co‐transformed with MdAMY1pro::GUS and either 35S::antiMdERF17 or 35S::antiMdbHLH149, treated with 10 mM ACC. GUS activity was quantified enzymatically and visualised by histochemical staining. Data represent mean ± SD of at least three biological replicates. Different letters indicate significant differences (*p* < 0.05, Student's *t*‐test). (e) Phenotype of ‘Luli’ apple fruits infiltrated with the indicated TRV‐based silencing vectors and 10 mM ACC. Scale bar, 2 cm. (f) RT‐qPCR analysis of *MdERF17*, *MdbHLH149* and *MdAMY1* expression in the infiltrated regions shown in (e). (g, h) Metabolic analysis of injection sites: Starch (g) and soluble sugar (fructose, sucrose, glucose) contents (h). Values are mean ± SD from at least three biological replicates (two similarly sized apples per replicate). Different letters indicate significant differences (*p* < 0.05, one‐way ANOVA).

To investigate the connection between ethylene signalling and the MdERF17–MdbHLH149–*MdAMY1* regulatory module, we treated MdAMY1pro::GUS, MdbHLH149pro::GUS and MdbHLH17pro::GUS transgenic apple calli with 10 mM ACC. GUS staining and quantitative activity assays revealed that ACC significantly enhanced *MdAMY1* promoter activity compared to the control, while MdbHLH149/MdERF17 promoter activity was repressed (Figure 6a–c). Then we generated transgenic apple calli expressing the MdAMY1pro::GUS reporter, together with antisense constructs targeting *MdERF17* or *MdbHLH149*. After treatment with the ethylene precursor ACC, GUS staining and quantitative activity assays revealed that ACC significantly enhanced *MdAMY1* promoter activity compared to the control (Figure 6d). Moreover, knockdown of either *MdbHLH149* or *MdERF17* under ACC treatment further increased GUS activity, with the strongest induction observed when both repressors were silenced simultaneously (Figure 6d). These results indicate that the MdERF17–MdbHLH149 module is influenced by ethylene signalling to suppress *MdAMY1* expression.

We next employed a VIGS‐based approach in ‘Luli’ apple fruits to dissect the functional hierarchy of this module during ethylene‐mediated starch degradation (Figure 6e). ACC treatment strongly downregulated *MdERF17* and *MdbHLH149* expression while upregulating *MdAMY1*, resulting in decreased starch content and elevated soluble sugars (fructose, sucrose and glucose). Silencing *MdAMY1* in ACC‐treated fruits attenuated the ethylene‐mediated conversion of starch into soluble sugars (Figure 6f–h). Further functional analyses showed that silencing *MdbHLH149* and *MdAMY1* under ACC treatment further reduced transcript levels of both *MdbHLH149* and *MdERF17*, enhanced *MdAMY1* expression, and intensified the metabolic phenotype—lower starch and higher soluble sugars. Similarly, silencing *MdERF17*, *MdbHLH149* and *MdAMY1* in the presence of ACC resulted in the greatest release of transcriptional repression on *MdAMY1* and the most pronounced stimulation of starch‐to‐sugar conversion (Figure 6f–h).

Collectively, these data establish that ethylene promotes starch degradation by repressing *MdERF17* and *MdbHLH149*, thereby relieving their inhibition of *MdAMY1* transcription and activating the sugar conversion pathway.

## 3 Discussion

Fruit ripening is a highly coordinated developmental process in which the conversion of starch to soluble sugars is critical for quality attributes (Klee and Giovannoni 2011; Zhang and Hao 2020). Although ethylene is a well‐established regulator of ripening in climacteric fruits (Adams et al. 2004), its specific role in controlling starch metabolism remains incompletely understood. Here, we report that ethylene promotes starch degradation in apple by inactivating a transcriptional repression module composed of MdERF17 and MdbHLH149, thereby unleashing the expression of the cell wall‐localised amylase gene *MdAMY1* (Figure 6). AMY serves as a key enzyme responsible for starch degradation in plants (Figure 2; Seung et al. 2013; Monroe and Storm 2018). In this study, using an integrated approach combining transcript profiling, in vitro enzyme assays, and phenotypic analysis of *MdAMY1*‐overexpressing and silenced apple, we demonstrated that *MdAMY1* is upregulated during fruit ripening and functions as a positive regulator of starch degradation (Figures 1 and 2). Subcellular localization assays further revealed that MdAMY1 localises to the cell wall (Figure 2b), a pattern distinct from the typical plastid/cytoplasmic distribution of most plant amylases (Chen et al. 2025). It is worth noting that certain α‐amylases in rice also exhibit dual localization in the cell wall and amyloplasts (Chen et al. 1994, 2004), suggesting that compartment‐specific localization of amylases may underlie specialised functional roles in starch mobilisation.

Beyond the role of *MdAMY1* as an executor of starch breakdown, we provide evidence that the conversion of starch to soluble sugars is not a passive process but is under active transcriptional restraint. An inhibitory module consisting of MdERF17, MdbHLH149 and *MdAMY1* tightly regulates this metabolic transition (Figure 5j–m). While previous research on fruit ripening has largely emphasised transcriptional activators—such as SlLOB1 in tomato (Song et al. 2023), MdEAEL1 in apple (Li, Liu, et al. 2025), and ethylene‐induced MdNAC14, MdMYB2 and MdNTL9 that enhance softening through activation of *MdHb1* (Zhao et al. 2025). Our research discovered that the expression of *MdAMY1* decreased in both the overexpressing *MdERF17* and *MdbHLH149* calli (Figure S9), which suggests that the MdERF17‐MdbHLH149 serves as a crucial transcriptional repressor module for starch degradation in apple. This finding unveils a previously underappreciated layer of transcriptional repression in the regulatory network controlling fruit maturation.

During early fruit development, soluble sugar levels remain low, and photoassimilates are increasingly partitioned into starch stored in plastids (Zhang et al. 2010; Li et al. 2016). Premature or excessive starch degradation can compromise fruit structural integrity and stress tolerance (Chen et al. 2022). During the stage of fruit development, *MdERF17* and *MdbHLH149* were highly expressed; the MdERF17–MdbHLH149 complex acts as a ‘brake’ that maintains starch reserves by suppressing *MdAMY1* expression, thereby ensuring stable energy storage until ripening signals such as ethylene are perceived (Figure S10). Upon ethylene induction, this repression is alleviated, enabling *MdAMY1*‐mediated starch degradation and subsequent sugar accumulation (Figure 6).

In the starch metabolic pathway of fruits, starch is first degraded into glucose. A portion of this glucose is then converted into sucrose via enzymatic reactions involving sucrose synthase (SUS) and sucrose phosphate synthase (SPS), utilising fructose as a substrate. Glucose, fructose and sucrose are maintained in a dynamic equilibrium and can be interconverted through various enzymatic steps (Yu et al. 2022). Illustrating this regulatory complexity, MdbZIP44 has been shown to promote starch accumulation in apple by repressing *Mda‐GP2* expression while reducing glucose levels (Cao et al. 2024). Similarly, in *SlVI* knockout fruits, sucrose accumulation triggers negative feedback that downregulates the starch degradation genes *BAM3* and *AMY2* (Wu et al. 2025). Furthermore, overexpression of *MdbHLH3* enhances photosynthetic efficiency and carbohydrate content in apple leaves, and significantly promotes carbohydrate accumulation in fruits by optimising the carbohydrate partitioning mechanism between source and sink (Yu et al. 2021). Consistent with these observations, our data show that shifts in starch abundance in both mature apple fruit and *MdAMY1*‐engineered lines are tightly coupled to coordinated changes in glucose, fructose and sucrose levels (Figures 1c,f and 2i,j), underscoring the tightly coupled nature of starch‐sucrose metabolic dynamics. These findings highlight the precise coordination in plants between energy allocation and developmental progression. Consequently, the regulatory mechanism uncovered here not only elucidates a key aspect of carbohydrate metabolism during ripening but also directly informs strategies for improving fruit quality and optimising harvest timing in apple.

**FIGURE 7:**
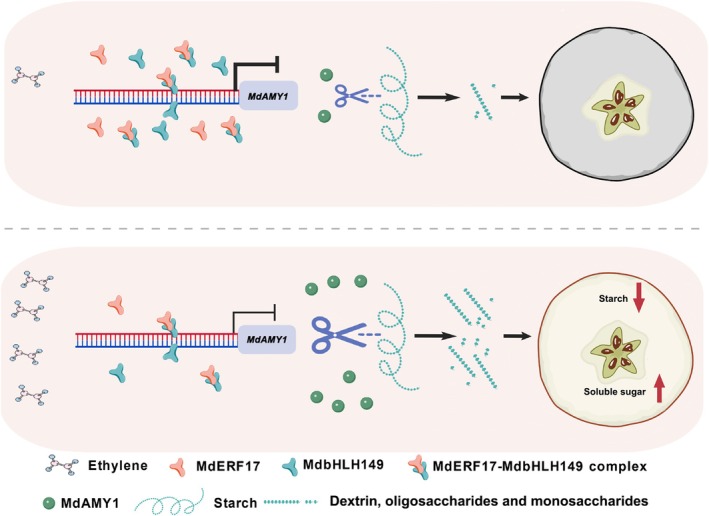
Proposed model for ethylene‐mediated regulation of starch degradation via the MdERF17–MdbHLH149 module in apple fruit. Under low ethylene conditions, elevated expression of *MdERF17* and *MdbHLH149* facilitates their physical interaction, which potentiates the transcriptional repression of *MdAMY1* by MdbHLH149. This results in suppressed *MdAMY1* expression and restricted starch degradation. By contrast, high ethylene levels downregulate both *MdERF17* and *MdbHLH149*, thereby relieving the repression on *MdAMY1*. The resulting induction of *MdAMY1* expression promotes starch breakdown and enhances the accumulation of soluble sugars during fruit ripening.

A combination of Y2H, pull‐down, BiFC and LUC complementation assays confirmed that MdERF17 physically interacts with MdbHLH149 to form a functional protein complex (Figure 5a–d). GUS activity assays further established MdbHLH149 as a transcriptional repressor of *MdAMY1*, whose repressive effect was enhanced by co‐expression with *MdERF17* (Figure 5i). Ethylene signalling was found to counteract this repression: treatment with 10 mM ACC significantly induced the expression of *MdAMY1*, whereas antisense‐mediated silencing of *MdERF17* or *MdbHLH149*—singly or together—markedly increased *MdAMY1* expression (Figure 6d). Consistent with this, ACC treatment reduced transcript levels of both *MdERF17* and *MdbHLH149* in ‘Luli’ apple fruits (Figure 6f). Although the precise mechanism by which ethylene suppresses this repressor complex remains unclear, we propose that ethylene may either directly downregulate their transcription or modulate their post‐translational modifications—such as ubiquitination or phosphorylation—through intermediary regulators, thereby influencing the stability or activity of the MdERF17‐MdbHLH149 complex. Collectively, our results establish a molecular link between ethylene signalling and starch catabolism and support a model in which ethylene promotes starch degradation and soluble sugar accumulation by dismantling the transcriptional repression of *MdAMY1* imposed by the MdERF17–MdbHLH149 module (Figure 7).

The experimental validation of MdERF17, MdbHLH149 and MdAMY1 as regulators of starch degradation (Figures 2g–j, 3g–j and 5e–h) positions them as promising targets for CRISPR/Cas9‐mediated molecular breeding in modern plant biotechnology. Suppressing inhibitory regulators such as MdERF17 and MdbHLH149 could accelerate starch breakdown and enhance fruit sweetness, thereby facilitating the development of sweeter cultivars. Conversely, enhancing their expression may improve postharvest quality by prolonging starch retention during storage and transport, enabling the breeding of varieties with superior storability and resilience under logistical stress. Beyond genetic regulation, numerous environmental factors—including light (Li, Yang, et al. 2025), temperature (Li, Ma, et al. 2025) and water availability (Yang et al. 2019)—critically influence fruit ripening and sugar accumulation. While conclusive evidence linking environmental signals to the direct regulation of starch degradation during fruit development remains limited, we hypothesize that such cues may directly modulate starch and sucrose metabolism, thereby shaping ripening dynamics. This hypothesis offers a theoretical framework for understanding regional variations in apple maturity and sweetness across diverse growing environments.

## 4 Methods

### 4.1 Plant Materials and Growth Conditions

Fruits of ‘Starking Delicious’ apple were collected from an orchard in Tianshui City, Gansu Province, China, at 100, 115 and 145 days after bloom (DAB). The mesocarp tissue (flesh, excluding peel and seeds) was dissected into small pieces, flash‐frozen in liquid nitrogen, and stored at −80°C for subsequent metabolic and transcriptomic analyses. All experiments included at least three biological replicates, with each replicate comprising two apples of uniform size and developmental stage.

For transient injection assays, fruits were harvested from mature ‘Starking Delicious’ (140 DAB) and ‘Luli’ (100 DAB) trees. The 35S::MdAMY1‐GFP, 35S::MdbHLH149‐GFP and 35S::MdERF17‐GFP constructs were introduced into ‘Orin’ apple calli via *Agrobacterium*‐mediated transformation. Transformed calli were subcultured repeatedly on Murashige and Skoog (MS) medium supplemented with 0.5 mg/L indole‐3‐acetic acid and 1.5 mg/L 6‐benzylaminopurine and maintained in darkness at 25°C to obtain stable transgenic lines.

Fruits from ‘Gala’, ‘Mato 1’, and their F_1_ hybrid population were obtained from the research orchard of the Shandong Fruit Research Institute, where all trees were maintained under standard horticultural and pest management practices.

‘GreenSleeve’ apple fruits were grown in the orchard of our research group. Fruit ripening commenced at approximately 90 days after bloom (DAB), and samples were collected at 30, 45, 60 and 90 DAB for gene expression analysis.

### 4.2 Virus‐Induced Gene Silencing in Apple Fruits

Gene‐specific primers were designed based on the apple genome GDDH13 (https://iris.angers.inra.fr/gddh13/; Table S1). PCR amplification was performed using in vitro ‘Royal Gala’ tissue cultures as template under the following conditions: initial denaturation at 98°C for 30 s, followed by 35 cycles of 98°C for 10 s, 58°C for 5 s and 72°C for 5 s. Amplified products were separated by 1.5% agarose gel electrophoresis, and target fragments were excised, purified and cloned into a T‐vector for sequencing.

Virus‐induced gene silencing was performed using TRV and pIR vectors. For gene silencing, partial fragments of *MdAMY1*, *MdbHLH149* and *MdERF17* were amplified using primer pairs MdAMY1‐F/R, MdbHLH149‐F/R and MdERF17‐F/R, respectively, and cloned into the TRV vector under a dual *35S* promoter. For sense infiltration, full‐length coding sequences of these genes were inserted into the pIR vector. All TRV constructs were introduced into *Agrobacterium tumefaciens* strain GV3101 and infiltrated into ‘Starking Delicious’ and ‘Luli’ fruits. After infiltration, apples were kept in darkness at 19°C for 7 days before analysis.

### 4.3 RNA Sequencing

Total RNA was extracted from ‘Starking Delicious’ apples harvested at 100, 115 and 145 DAB, with three biological replicates per stage (each comprising one fruit). RNA integrity was assessed by 1% agarose gel electrophoresis, and purity was determined using a NanoDrop spectrophotometer (IMPLEN). Sequencing libraries were constructed from 1 μg total RNA per sample using the NEBNext Ultra RNA Library Prep Kit for Illumina (NEB) according to the manufacturer's instructions. Briefly, mRNA was enriched using poly‐T oligo(dT) magnetic beads and fragmented in NEBNext First Strand Synthesis Reaction Buffer (5×) under elevated temperature. First‐strand cDNA was synthesized with random hexamer primers and M‐MuLV reverse transcriptase, followed by second‐strand synthesis using DNA polymerase I and RNase H. Double‐stranded cDNA was end‐repaired, adenylated, and ligated to NEBNext adapters. Fragments of 250–300 bp were selected using AMPure XP beads (Beckman Coulter) and amplified with Phusion High‐Fidelity DNA polymerase. Library quality was assessed on an Agilent Bioanalyzer 2100, and paired‐end sequencing (150 bp) was performed on an Illumina platform after cluster generation on the cBot system.

### 4.4 Bioinformatics Analysis

Differentially expressed genes (DEGs) were identified, and KEGG and GO enrichment analyses were performed using the OmicStudio cloud platform (https://www.omicstudio.cn/tool). The tertiary structure of MdAMY1 was predicted using Phyre2 (http://www.sbg.bio.ic.ac.uk/phyre2).

### 4.5 Scanning Electron Microscopy (SEM)

Apple pulp samples were prepared as described (Zhao et al. 2025). Critical point drying was performed using liquid CO_2_ in a Hitachi HCP‐2 dryer with temperature steps at 0°C and 20°C (20 min each), followed by 35°C for 15 min. Dried samples were manually divided, mounted on holders, sputter‐coated with gold using a JFC‐600 system, and observed under a JSM‐6610LV scanning electron microscope.

### 4.6 Histological Analysis

Tissues were fixed at 4°C for 5 h, rinsed with PBS, and embedded in Epon 812 resin via propylene oxide transition. Polymerisation was carried out at 36°C (12 h), 45°C (12 h) and 60°C (36 h). Semithin sections were prepared with a Leica RM2265 microtome, stained with schiff reagent in the dark for 10–20 min, rinsed with distilled water, and imaged under an Olympus BX51 light microscope.

### 4.7 RNA In Situ Hybridization

Tissue fixation and hybridization were performed as previously described (Wang et al. 2014). Fresh apple fruit samples were immediately fixed in FAA fixative for 12 h. Following dehydration through a graded series of ethanol solutions, the samples were cleared in xylene and embedded in paraffin wax. Sections of 8 μm thickness were cut using a Leica RM2235 microtome (Leica Microsystems, Wetzlar, Germany). After dewaxing and rehydration, the sections were hybridised overnight at 42°C with digoxigenin (DIG)‐labelled MdAMY1 and MdbHLH149 antisense RNA probes, which were synthesised in vitro using T7 RNA polymerase and the Roche DIG RNA Labeling Kit (Roche Diagnostics, Indianapolis, IN, USA). Post‐hybridization washes were performed sequentially with 2 × SSC and phosphate‐buffered saline (PBS). The sections were then incubated with 1% blocking reagent for 45 min, followed by incubation with the antibody working solution (3 μL anti‐DIG antibody +1 mL blocking reagent) for 2 h. A colorimetric reaction was initiated by the addition of the colour development solution (20 μL NBT/BCIP stock solution +1 mL NTM buffer) and allowed to proceed overnight in the dark. Finally, the sections were rinsed in NTM buffer to stop the reaction and observed under an optical microscope (Olympus BX53, Olympus Corporation, Tokyo, Japan).

### 4.8 Recombinant Protein Expression and Purification

The coding sequence of *MdAMY1* was cloned into pET‐32a and transformed into *Escherichia coli* BL21 (DE3). Protein expression was induced with 50 μM isopropyl‐β‐d‐thiogalactoside at OD600 ≈0.6, followed by incubation at 16°C for 20 h. Cells were harvested, resuspended in lysis buffer (50 mM NaH_2_PO_4_, 300 mM NaCl, 10 mM imidazole, pH 8.0), and lysed by sonication. After centrifugation, hemin chloride was added to the soluble fraction. MdAMY1‐His was purified by Ni‐NTA affinity chromatography and dialyzed against 50 mM NaH_2_PO_4_ and 300 mM NaCl (pH 8.0) at 4°C. Protein concentration was determined by the Bradford method. Subsequently, SDS‐PAGE was used to analyse protein bands, and ImageJ (v1.53) was employed for densitometric quantification. The specific procedures were as follows: Open the gel image using ImageJ and convert it to an 8‐bit grayscale image. Remove background shadows and set measurement parameters, then select protein bands for analysis. Statistical analysis was performed based on the resulting values.

### 4.9 In Vitro Enzyme Activity Assay

A 1% (w/v) soluble starch solution was prepared in citrate‐Na_2_HPO_4_ buffer (50 mM, pH 7.5) and gelatinized by heating in a boiling water bath for 10 min with stirring. The 600 μL reaction system contained 275 μL buffer, 300 μL starch solution, and 25 μL diluted enzyme. After pre‐incubation at 35°C for 10 min, the reaction was terminated by adding 300 μL of 3,5‐dinitrosalicylic acid (DNS) reagent. The mixture was vortexed, boiled for 10 min, cooled on ice, and centrifuged; absorbance of the supernatant was measured at 540 nm. Kinetic parameters were derived by fitting initial velocities to the Michaelis–Menten equation using GraphPad Prism 8.0.

### 4.10 Subcellular Localization

The coding sequences of *MdAMY1* and *MdbHLH149* were fused in‐frame to GFP under the *35S* promoter (35S::MdAMY1‐GFP and 35S::MdbHLH149‐GFP) as previously described (Wang, Zhao, et al. 2022). Constructs were transiently expressed in tobacco leaves via *Agrobacterium* infiltration. After 2 days, epidermal cells were stained with propidium iodide or DAPI for 5 min and imaged using a Zeiss LSM 880 confocal microscope (LSM 880, Carl Zeiss, Meta) with excitation at 488, 405 and 561 nm.

### 4.11 Chromatin Immunoprecipitation‐Quantitative Polymerase Chain Reaction (ChIP‐qPCR)

ChIP‐qPCR assays were conducted on 35S‐GFP and MdnHLH149‐GFP transgenic apple calli tissues following the previously protocol described (Zhang et al. 2025). Immunoprecipitation was performed using an anti‐GFP antibody (10 μg/mL; Beyotime, Suzhou, China), and all primer sequences used in this study are listed in Table S1.

### 4.12 Yeast One‐Hybrid (Y1H)

The full‐length coding sequences of *MdNTL9*, *MdNAC14*, *MdMYB2*, *MdHSF24*, *MdERF17*, *MdWRKY53*, *MdbHLH149*, *MdLBD4*, *MdERF102*, *MdMYC2* and *MdAUX28* were individually cloned into the pGAD424 vector, and the promoter region of *MdAMY1* was inserted into the pHis2 vector. Yeast one‐hybrid assays were subsequently conducted with 3‐amino‐1,2,4‐triazole as a selective agent, following the previously described protocol (Hu et al. 2025).

### 4.13 Electrophoretic Mobility Shift Assays (EMSA)

EMSA was performed as previously described (Wang, Xiao, et al. 2025). Biotin‐labelled probes were detected using a Biotin Labeling Kit and a LightShift Chemiluminescent EMSA Kit (Thermo Scientific, Waltham, MA, USA). The MdbHLH149 protein was incubated with a biotin‐labelled oligonucleotide probe derived from the *MdAMY1* promoter in binding buffer at room temperature for 20 min. To confirm binding specificity, unlabeled (cold) probes were included in competition assays.

### 4.14 Dual‐Luciferase Assay and β‐Glucuronidase (GUS) Analysis

Transient expression analysis was conducted in tobacco leaves. The *MdAMY1* promoter was cloned into the pGreenII 0800‐Luc vector, while MdbHLH149 was inserted into the pGreenII 62‐SK vector. *A. tumefaciens* strain LBA4404 was used for transformation and leaf infiltration. Fluorescence signals were captured using a live imaging system. The GUS reporter construct, containing the *MdAMY1* promoter sequence, was prepared as previously described (Hu et al. 2017).

### 4.15 Yeast Two‐Hybrid Assay, GST Pull‐Down Assay, Bimolecular Fluorescence Complementation Assay and Luciferase Complementation Assay

Y2H was performed using the Matchmaker Gold System (Clontech). *MdERF17* and *MdbHLH149* were cloned into pGAD424 and pGBT9, respectively, and co‐transformed into yeast strain AH109. Interactions were tested on SD/−Leu/−Trp/–His/−Ade medium following the previously described protocol (Wang, Li, et al. 2022).

For GST pull‐down, MdbHLH149 and MdERF17 were expressed as GST and His fusions, respectively. Purified proteins were incubated and pulled down as previously described (Wang et al. 2023).

BiFC and Transient dual‐luciferase assays were performed with the methods as described (Wang et al. 2024). For BiFC, MdERF17 and MdbHLH149 were fused to nYFP and cYFP, expressed in *N. benthamiana* leaves via Agrobacterium infiltration, and imaged by two‐photon confocal microscopy (Zeiss LSM Meta). For dual‐luciferase complementation, genes were cloned into nLUC and cLUC vectors, expressed in tobacco, and imaged using a live imaging system (Xenogen).

### 4.16 I_2_‐KI Staining and Ethylene Measurement

Cross‐sections of apple fruits were stained with I_2_–KI, and ethylene release was quantified as described (Qiao et al. 2026).

### 4.17 Metabolite Quantification

Starch was determined using commercial kits (Solarbio, BC0700). Weigh precisely 0.1 g of the sample and introduce 0.6 mL of Reagent 1. Conduct the extraction at 80°C in a water bath for 30 min. Centrifuge the mixture at 3000*g* at room temperature for 5 min. Discard the supernatant and retain the precipitate. Add 0.3 mL of distilled water to the precipitate and place it in a boiling water bath for gelatinization for 15 min. After cooling, add 0.6 mL of Reagent 2 and perform the extraction in the boiling water bath for 15 min. After cooling again, centrifuge at 8000*g* at room temperature for 15 min. Transfer the supernatant to the reaction reagent and measure the absorbance value at 620 nm for the calculation of the starch content.

Soluble sugar was determined using commercial kits (Solarbio, BC0035). Weigh approximately 0.1 g of the sample, add 1 mL of distilled water, and incubate it in a boiling water bath for 10 min. After cooling, conduct a centrifugation at 8000 g at room temperature for 10 min. Transfer the supernatant to a 10 mL test tube. Dilute it with distilled water to a final volume of 10 mL, and shake it thoroughly for storage. Add the supernatant to the reaction reagent and measure the absorbance value at 620 nm to calculate the soluble sugar content.

For the quantification of fructose, sucrose and glucose, approximately 0.1 g of sample was weighed, mixed with 5 mL of 80% ethanol, and incubated in a water bath at 80°C for 30 min. After cooling, the mixture was centrifuged at 5000 rpm for 5 min. The supernatant was evaporated to dryness in an oven, re‐dissolved in 1 mL of water, filtered through a 0.45 μm membrane, and analysed using a high‐performance liquid chromatography (HPLC) system.

### 4.18 Statistical Analyses

All experiments included at least three replicates. Data are presented as mean ± SD. Differences between two groups were assessed by Student's *t*‐test; multiple comparisons were analysed by one‐way ANOVA with post hoc tests. Significance was set at *p* < 0.05.

## Author Contributions

D.‐G.H. conceived and designed the study. F.X., C.‐K.W., J.‐C.Z., X.‐Y.J., Y.X., W.‐J.Z., J.‐C.M. and W.‐Y.W. performed the experiments and analysed the data. C.‐K.W., F.X., and D.‐G.H. wrote the paper. All authors discussed the results and commented on the manuscript.

## Funding

This work was supported by grants from the Taishan Scholar Project Special Funds of China (Grant No. tstp20250723), National Key Research and Development Program of China (2023YFD2301000, 2022YFD2100100), National Natural Science Foundation of China (32572986), Key Research and Development Program of Shandong Province (2023CXGC010709), and Shandong Postdoctoral Science Foundation (SDZZ‐ZR‐202501248).

## Conflicts of Interest

The authors declare no conflicts of interest.

## Supporting information


**Data S1:** Annotation of differentially expressed genes detected in the fruits of ‘Starking Delicious’ apple at the 100 vs. 115 (DAB) starch and sucrose metabolic pathways via RNA‐seq.

**Data S2:** Annotation of differentially expressed genes detected in the fruits of ‘Starking Delicious’ apple at the 115 vs. 145 (DAB) starch and sucrose metabolic pathways via RNA‐seq.

**Data S3:** Annotation of differentially expressed genes detected in the fruits of ‘Starking Delicious’ apple treated with 1‐MCP via RNA‐seq.

**Data S4:** Annotation of differentially expressed transcription factors detected in the fruits of ‘Starking Delicious’ apple at 100, 115 and 145 (DAB) via RNA‐seq.

**Appendix S1:** pbi70561‐sup‐0005‐AppendixS1.docx.

## Data Availability

All the data are included in the manuscript and the Supporting Information. Sequence data from this article can be found in the APPLE GENOME AND EPIGENOME (https://iris.angers.inra.fr/gddh13/) under accession number: MD08G1101700 (MdAMY1), MD16G1262200 (MdbHLH149) and MD15G1221100 (MdERF17).
