# Supplementary material for: The MdERF17–MdbHLH149 Module Mediates Ethylene‐Induced Starch Degradation Through the Transcriptional Repression of α‐Amylase MdAMY1 in Apple
Source: Plant Biotechnol J. 2026 Jan 23;24(5):3141–57. doi: 10.1111/pbi.70561 (PMC13110154; doi:10.1111/pbi.70561)
Supplement: Supplementary file 5 — Appendix S1: pbi70561‐sup‐0005‐AppendixS1.docx. [file PBI-24-3141-s004.docx]

**Supplemental Figures**


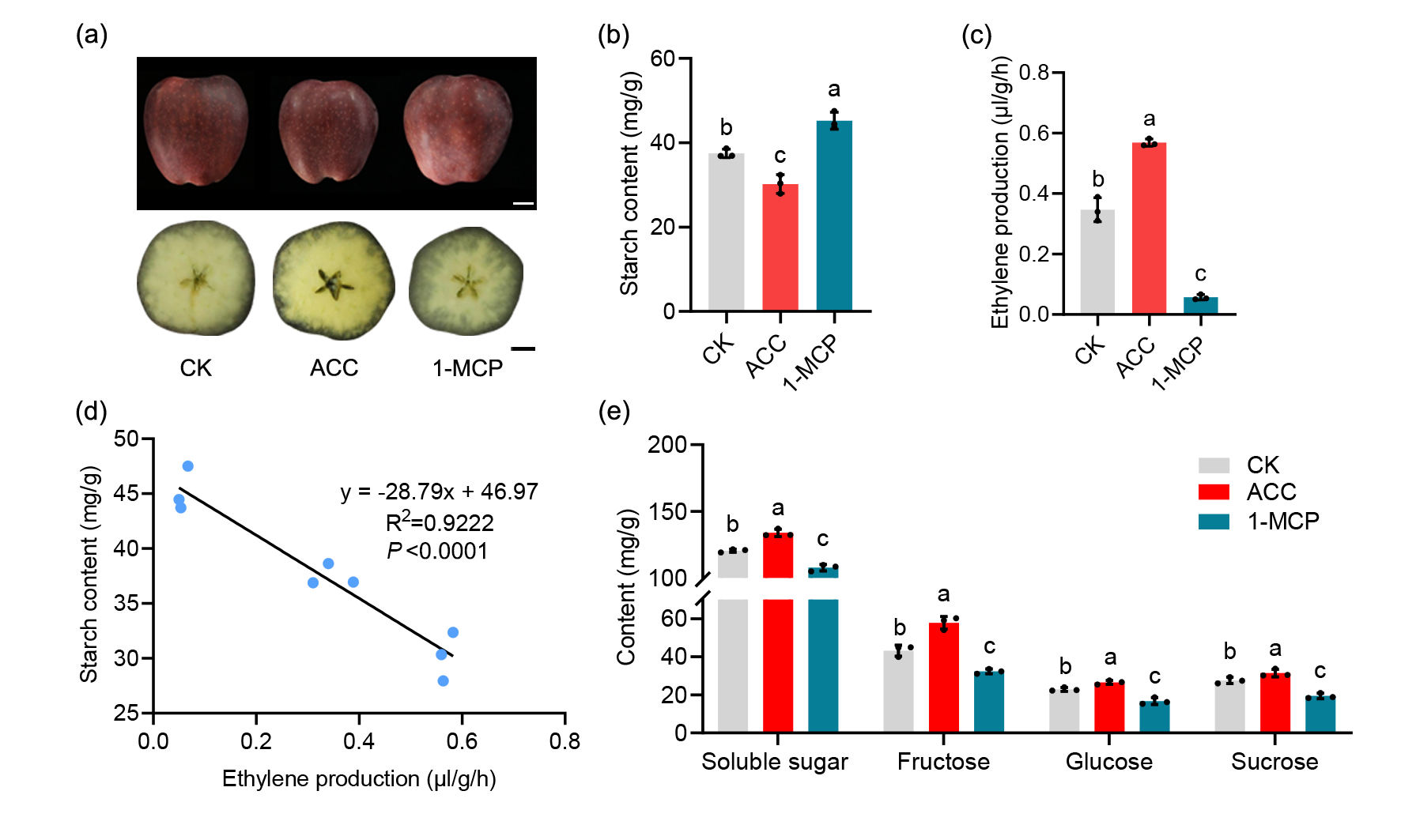


**Figure S1. Ethylene promotes the conversion of starch into soluble sugars in apple fruit.**

**(a)** KI-I_2_ staining results of apple fruits treated with 10 mM ACC and 1-MCP. Scale bar, 2 cm.

**(b,c)** Quantitative analysis of starch content (b) and ethylene emission rate (c) in apples subjected to 10 mM ACC (concentration) or 1-MCP (concentration) treatments as shown in (a).

**(d)** The correlation between starch content and ethylene release in (a).

**(e)** Soluble sugars, fructose, sucrose, and glucose content in apples subjected to 10 mM ACC (concentration) or 1-MCP (concentration) treatments as shown in (a).

In (b, c and e), statistical analysis was performed using one-way ANOVA. Different lowercase letters denote statistically significant differences at *P* < 0.05. Data are presented as mean ± standard deviation (SD) from at least three independent biological replicates.

**
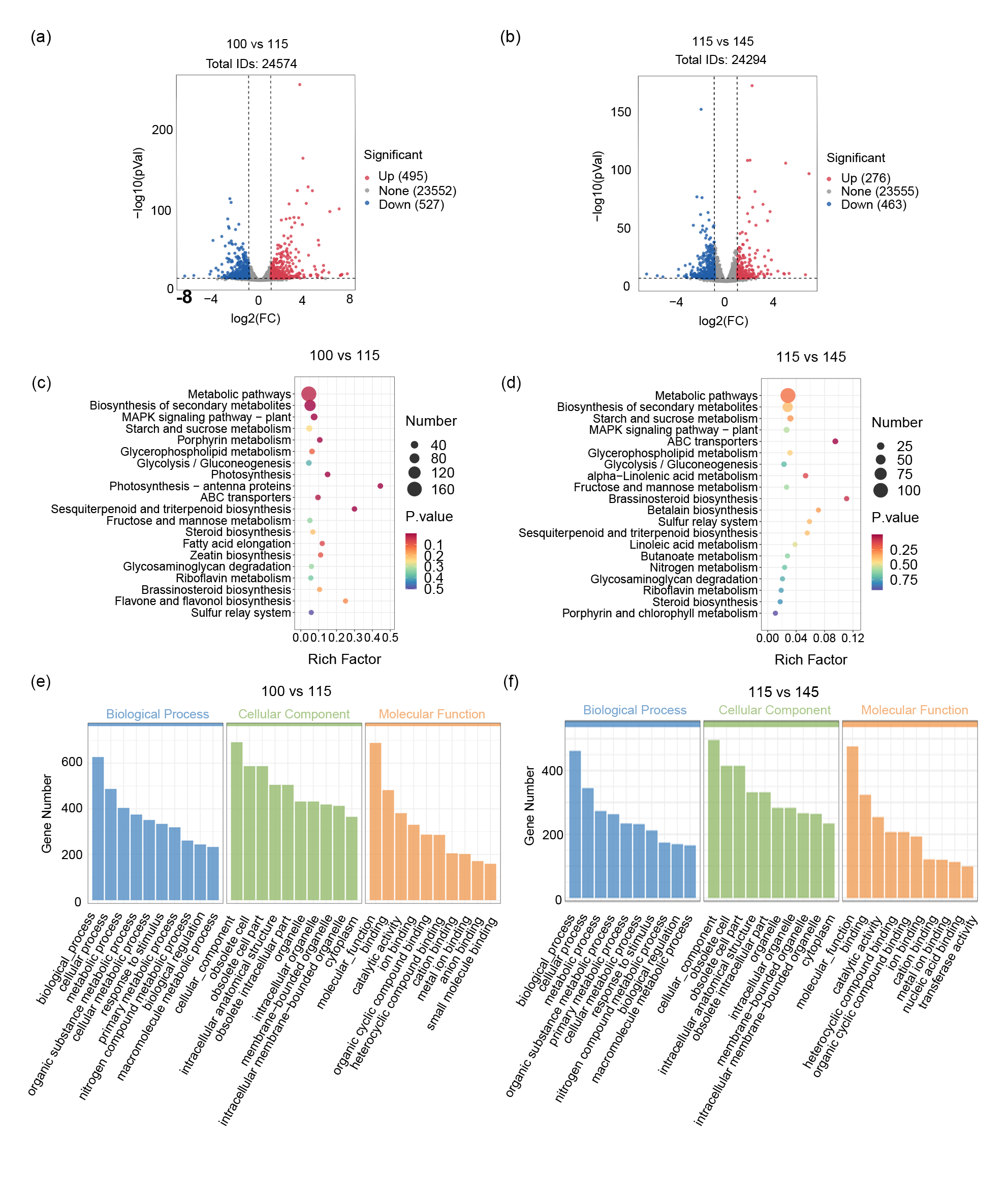
Figure S2. Transcriptomic analysis of apple fruits at three developmental stages: 100, 115, and 145 days after bloom (DAB).**

**(a,b)** Volcano plots illustrating differentially expressed genes (DEGs) with a |Log2(FC)| ≥ 1 and adjusted *p*-value < 0.001, comparing transcriptomes between 100 and 115 DAB (a) and between 115 and 145 DAB (b).

**(c,d)** KEGG pathway enrichment analysis of DEGs identified in the respective comparisons: 100 vs. 115 DAB (c) and 115 vs. 145 DAB (d). Pathways are ranked according to the number of associated DEGs, with the top 20 most enriched pathways displayed.

**(e,f)** Gene Ontology (GO) enrichment analysis of DEGs for the corresponding comparisons—100 vs. 115 DAB (e) and 115 vs. 145 DAB (f).

**
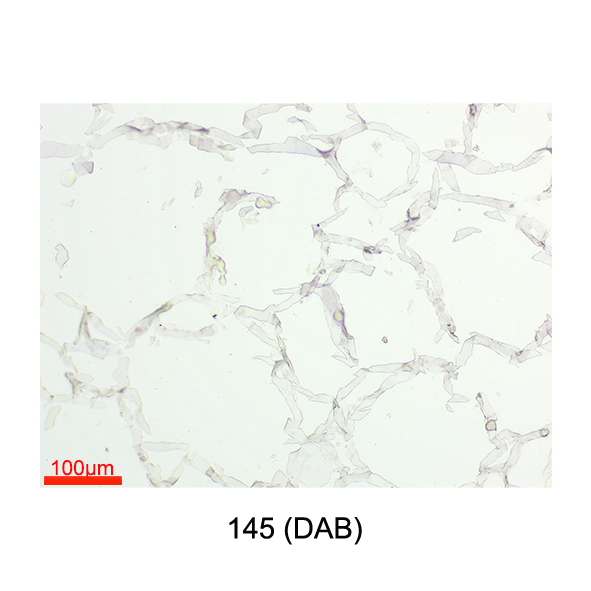
Figure S3. RNA *in situ* hybridization analysis of *MdAMY1.***

The expression of *MdAMY1* in apple fruits was examined using RNA *in situ* hybridization, with the sense strand probe serving as a negative control. Scale bar, 100 μm.


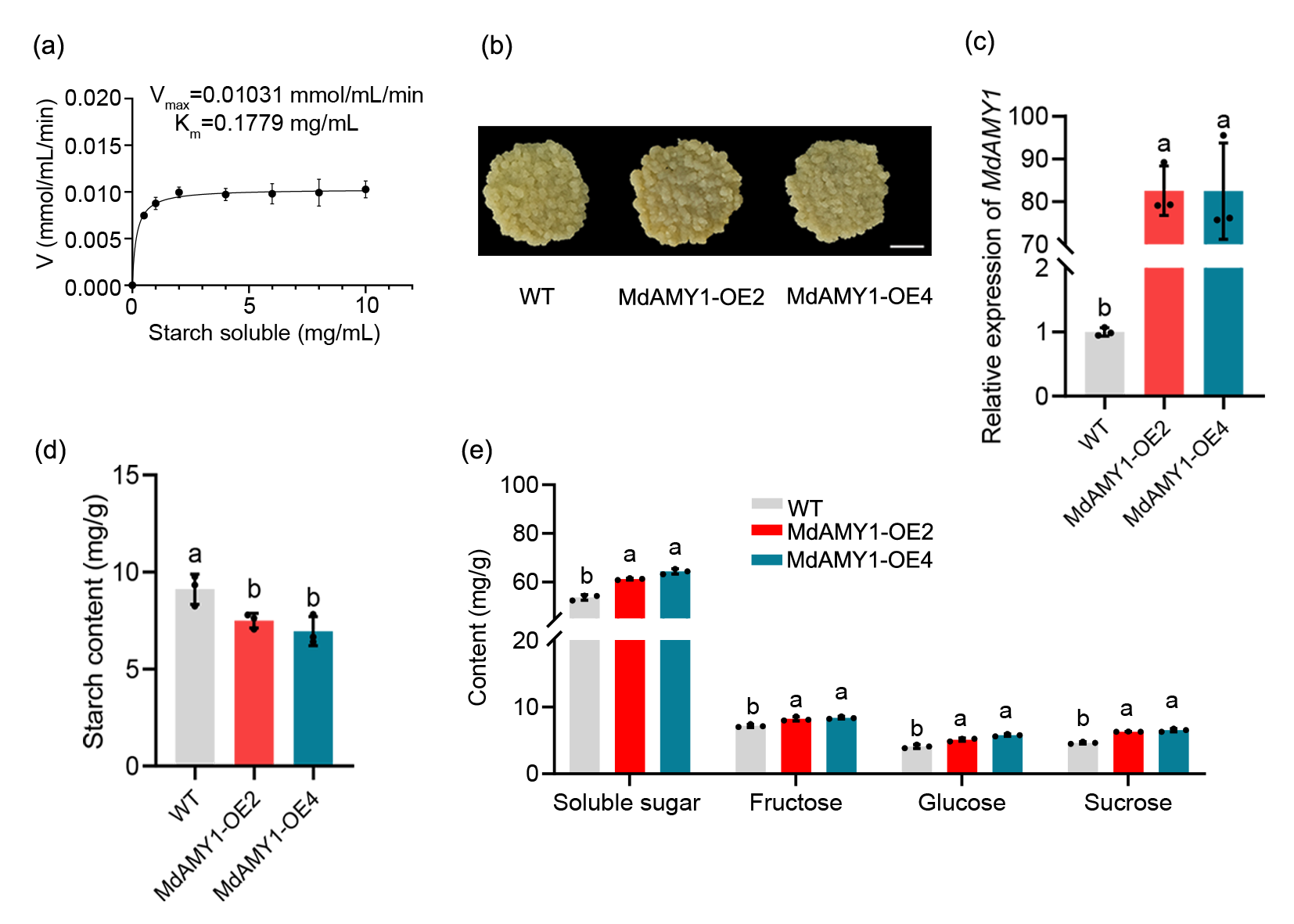


**Figure S4. *MdAMY1* promotes the degradation starch into soluble sugars in apple calli.**

**(a)** Enzyme activity curve of recombinant MdAMY1-His protein. Data are presented as mean ± standard deviation (SD) from three independent experiments (*n* = 3). Kinetic parameters were determined using nonlinear regression analysis with GraphPad Prism 8.0 software.

**(b)** Wild-type and *MdAMY1*-overexpressing apple callus lines. Scale bar, 2 cm

**(c)** Relative expression of *MdAMY1* in wild-type and MdAMY1-OEs calli

**(d)** Starch content in wild-type and *MdAMY1*-overexpressing calli.

**(e)** Content of soluble sugars, fructose, glucose, and sucrose, in wild-type and *MdAMY1*-overexpressing calli.

Data (c-e) are presented as mean ± standard deviation (SD) from at least three biological replicates, with each replicate comprising two apples of comparable size. Statistical significance was determined by one-way ANOVA; different lowercase letters denote significant differences at *P* < 0.05.


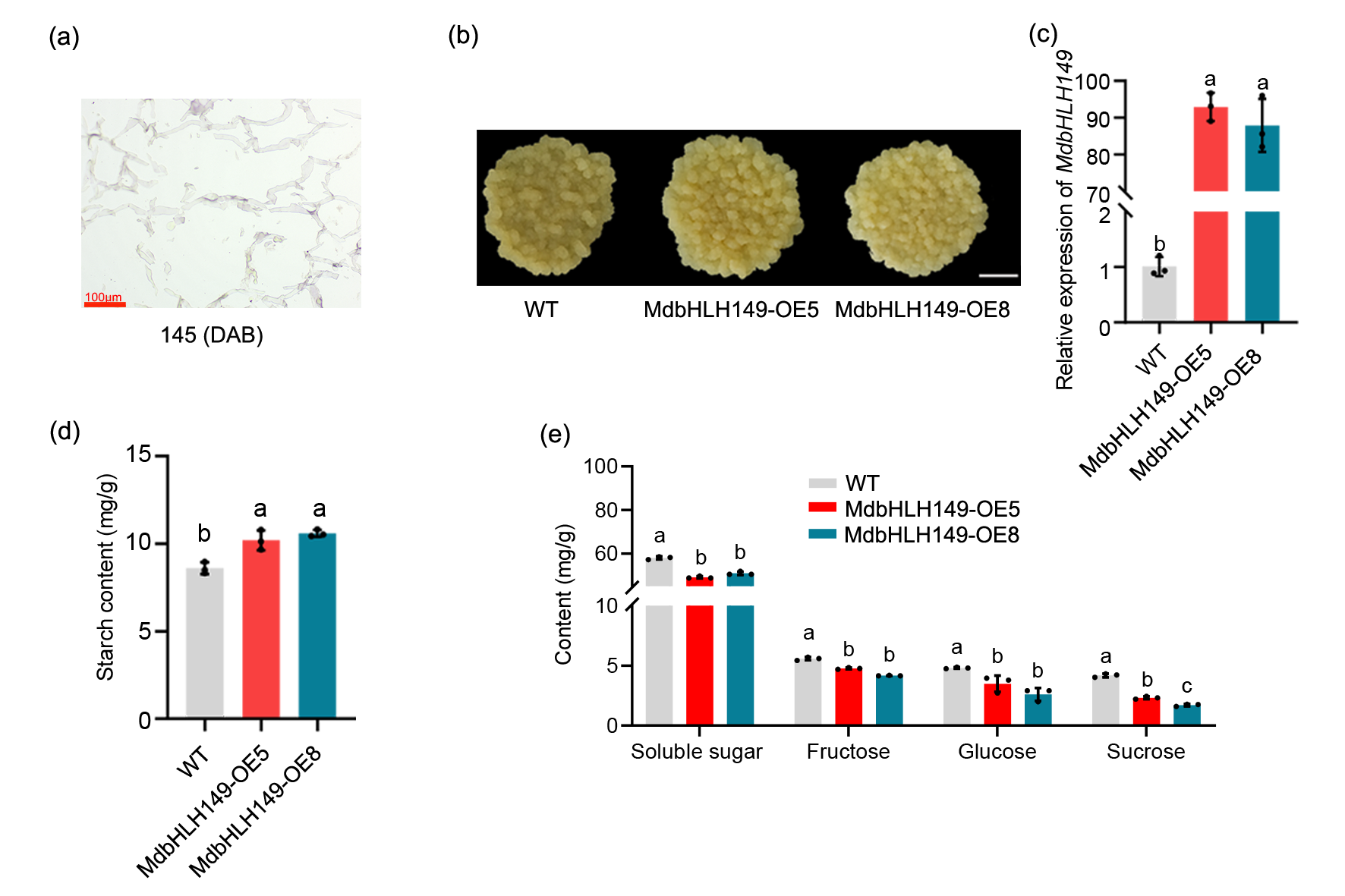


**Figure S5. *MdbHLH149* inhibits the conversion of starch into soluble sugars in apple calli.**

**(a)** Expression of *MdbHLH149* in apple fruits was analyzed by RNA *in situ* hybridization, with the sense strand probe used as a negative control. Scale bar, 100 μm.

**(b-e)** Phenotype (b), relative expression of *MdbHLH149* (c), starch content (d) and sugars levels (e) in Wild-type and *MdbHLH149*-overexpressing calli.

Data (c-e) are presented as mean ± standard deviation (SD) from at least three biological replicates, with each replicate comprising two apples of comparable size. Statistical significance was determined by one-way ANOVA; different lowercase letters denote significant differences at *P* < 0.05.


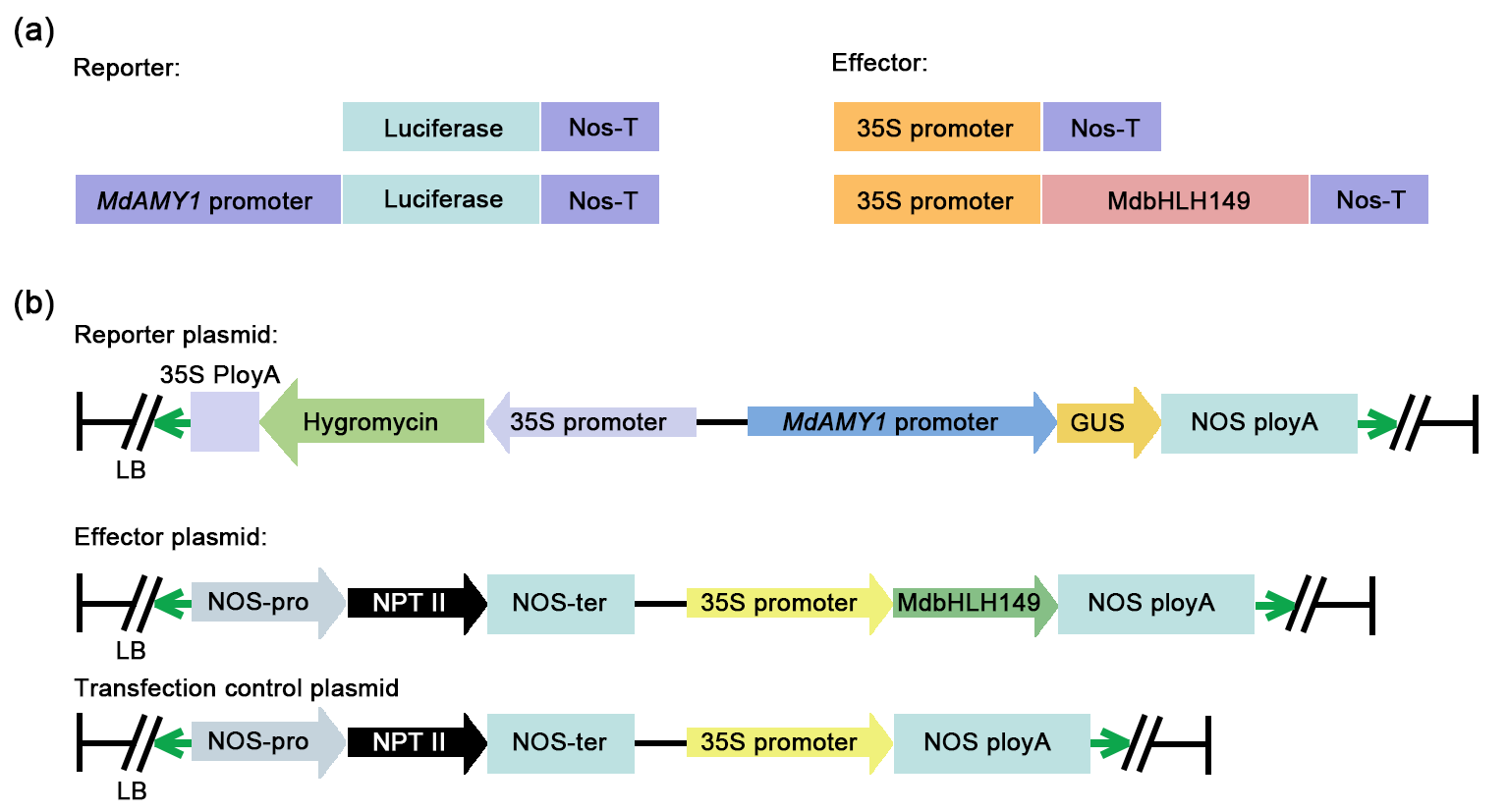


**Figure S6. Schematic representation of vectors in (bio) luminescence/GUS experiments.**

(a) Schematic representation of the LUC reporter vectors (MdAMY1pro::LUC) and the effector vector (35S::MdbHLH149).

(b) Schematic representation of GUS reporter vectors (MdAMY1pro::GUS) and effector vector (35S::MdbHLH149).


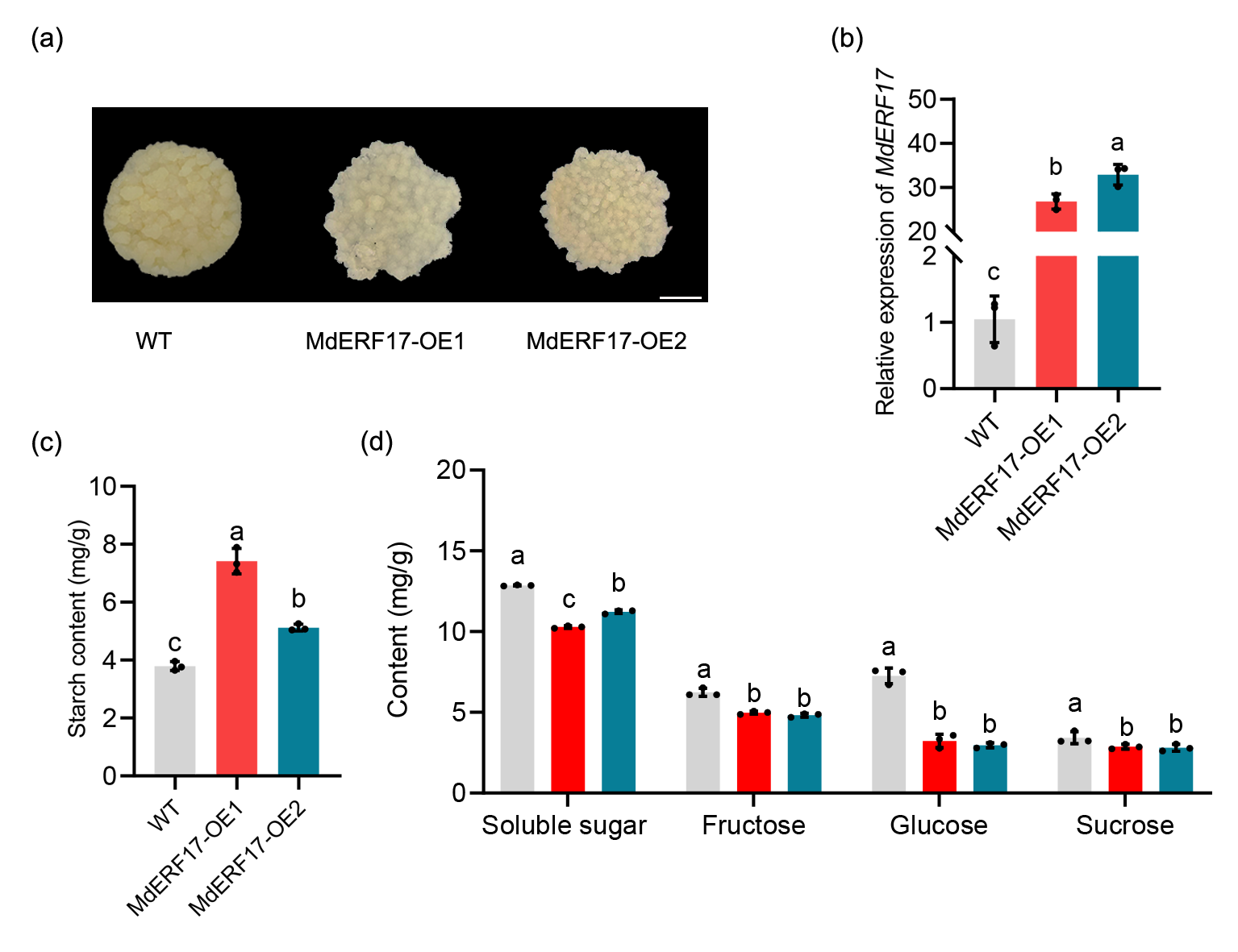


**Figure. S7. *MdERF17* inhibits the conversion of starch into soluble sugars in apple calli.**

**(a-d)** Phenotype (a), relative expression of *MdERF17* (b), starch content (c) and sugars levels (d) in Wild-type and *MdERF17*-overexpressing calli.

Data are presented as mean ± standard deviation (SD) from at least three biological replicates, with each replicate comprising two apples of comparable size. Statistical significance was determined by one-way ANOVA; different lowercase letters denote significant differences at *P* < 0.05.


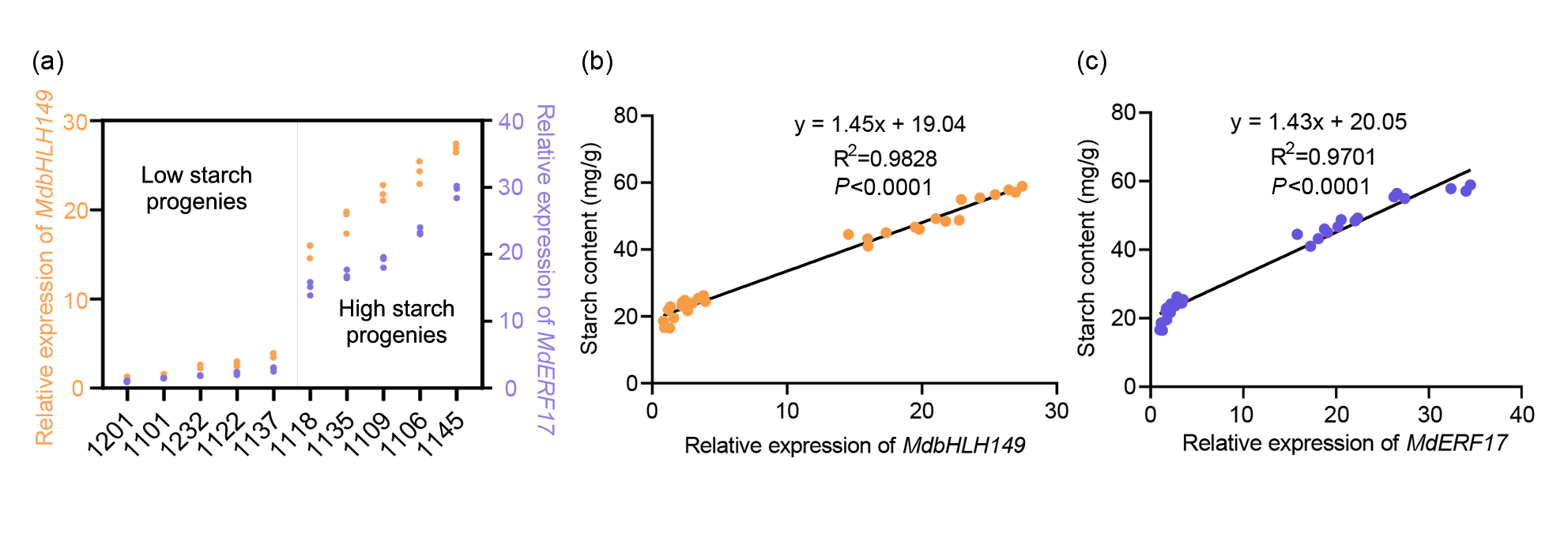


**Figure. S8. The relative expression of *MdbHLH149* and *MdERF17* were positively correlated with starch content in the 'Gala' × 'Mato 1' F₁ hybrid population.**

**(a)** The relative expression of *MdbHLH149* and *MdERF17* of apple fruit in the hybrid population.

**(b-c)** Correlation analysis between the content of starch and relative expression of *MdbHLH149* (b) and *MdERF17* (c) of fruits in 10 progenies.


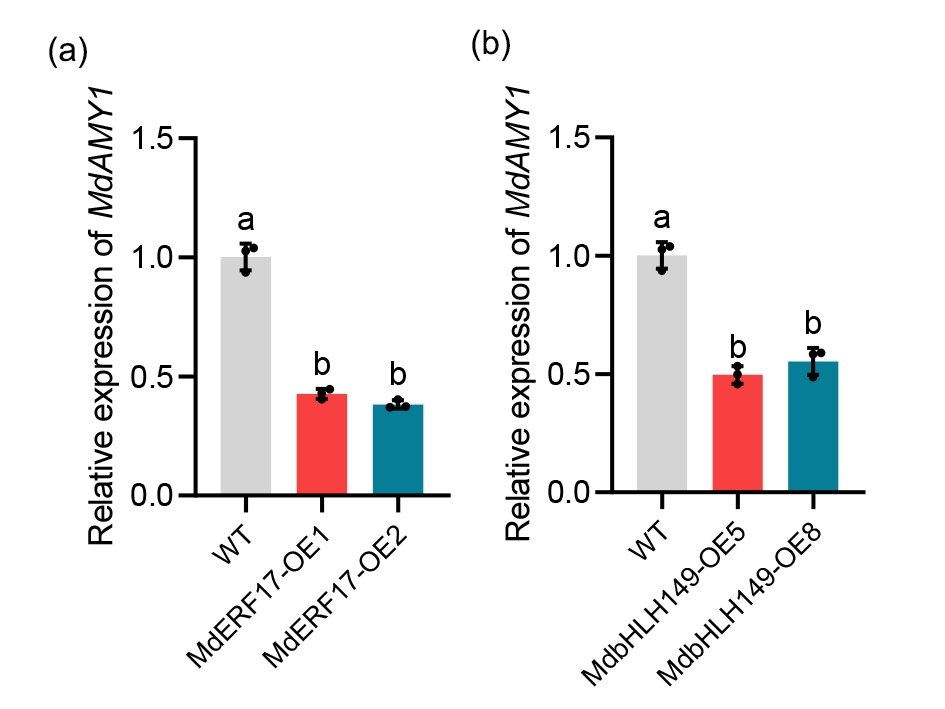


**Figure S9. Relative expression of *MdAMY1* in calli.**

Relative expression of *MdAMY1* in MdERF17-overexpressing calli (a) and MdbHLH149-overexpressing calli (b).

Data are presented as mean ± standard deviation (SD) from at least three biological replicates, with each replicate comprising two apples of comparable size. Statistical significance was determined by one-way ANOVA; different lowercase letters denote significant differences at *P* < 0.05.

**
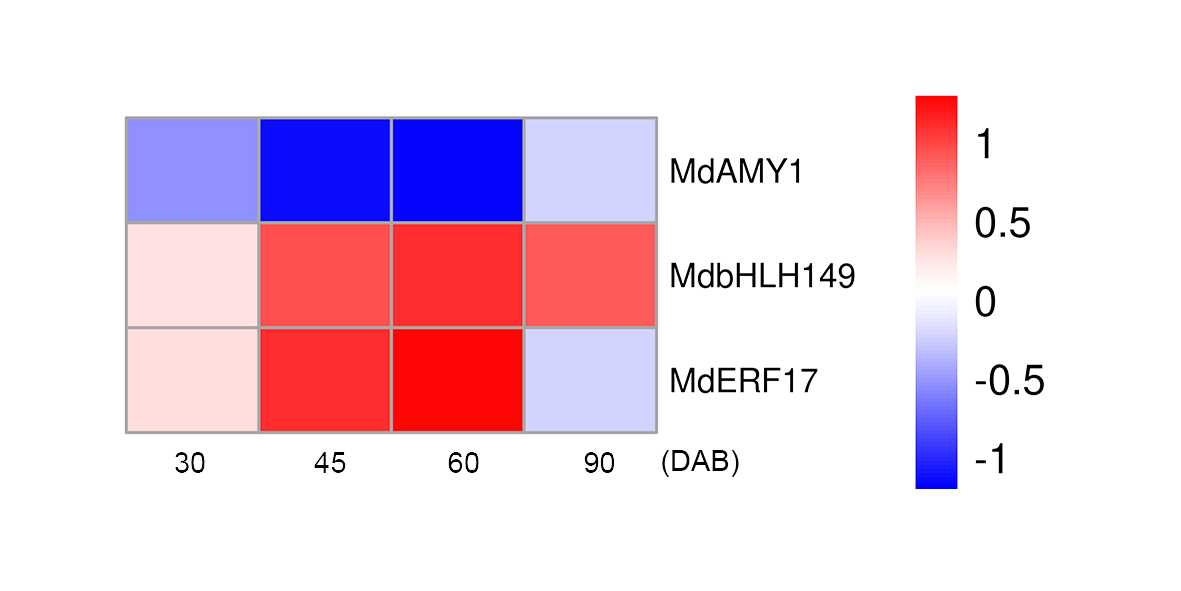
**

**Figure S10. The relative gene expression levels of *MdAMY1*, *MdbHLH149* and *MdERF17* during the 'Greensleeves' apple fruit development period.**

**Table S1. List of primers used in this study.**

| **Primers name** | **Primers sequence (5’ → 3’)** |
| --- | --- |
| MdAMY1-F | ATGAACTTCCTCCTTTCCTTCT |
| MdAMY1-R | TCAGTTTCTCTCCCACACAGC |
| MdMYC2-F | GCCAAGAACCGACCTCCAAAT |
| MdMYC2-R | GCCGAACAAGAGTACCGCAAG |
| MdAUX28-F | CCCCTCCTTCCTAATCCTTCC |
| MdAUX28-R | CAACCTGCTCAATCCATCTGC |
| MdERF102-F | ATGGCTTTAGATCAGGTCTCGG |
| MdERF102-R | TCATACGACCATAAGCTGTGGAC |
| MdERF17-F | ATGGTGAAGCAGACGATTGGG |
| MdERF17-R | CTAGAAATTCCAGAGGAAAGACTCCT |
| MdbHLH149-F | TTTCTGAACAAATGGATAAAGGGTC |
| MdbHLH149-R | GGGCACCTTAAAGTTACAGCTACAT |
| MdWRKY53-F | GCTCACGAATCTCCTTGGGTT |
| MdWRKY53-R | AACTTATTCTTATTCTCAAAGACCGACTA |
| MdLBD4-F | ACTGCCTACTGTTGGGGATTG |
| MdLBD4-R | TCGCCTTGGTCACTGTCATCGT |
| MdMYB2-F | ATGTATTTGGGGATGATGGCAG |
| MdMYB2-R | GCTTTGCCTGGTGGTGTTAATAAAG |
| MdNAC14-F | ATGGCTGCCAATGGAATCCACT |
| MdNAC14-R | TTTAATCAATTGAGATATCTTCTTTG |
| MdNTL9-F | ATGAGCAGCAGCAGCAGCAGCA |
| MdNTL9-R | ATTAAAGCAATACTTACTAGAGATCAG |
| MdHSF24-F | ATGGCGCAAAGGTCCGTT |
| MdHSF24-R | GTTACACACCTTCCCACTCCTC |
| MdAMY1pro-F | ACTAATGTTATGGGAATGCGTGC |
| MdAMY1pro-R | AAGGAAAGGAGGAAGTTCATGGT |
| qMdAMY1-F | AGTTGGACCTTGGAGACCTT |
| qMdAMY1-R | CTCCCACACAGCAAAGTCTT |
| qMdbHLH149-F | GACCAACTTAGACAGAAAGAAGGC |
| qMdbHLH149-R | GTAGCATTGGCTATGACGGC |
| qMdERF17-F | GGTTGTTTGCGGGTTTTGAT |
| qMdERF17-R | ACTGACCGTCCAAGTTCTCT |
| 18S-F | ACACGGGGAGGTAGTGACAA |
| 18S-R | CCTCCAATGGATCCTCGTTA |
| MdAMY1-F(Chip-P1) | GAGTTTCAATATCCGCCAGTCT |
| MdAMY1-R(Chip-P1) | AGAGAAGCTAGACACTTGGTG |
| MdAMY1-F(Chip-P2) | GCCGAGTCTGCCATTTTCTA |
| MdAMY1-R(Chip-P2) | GTGCCTTTATACTCGTATATCTCGT |
| MdAMY1-F(Chip-P3) | CCTCACGAACTCTGACAATGAA |
| MdAMY1-R(Chip-P3) | GTTGATCAGTGCTGGGACTA |
| MdAMY1-F(Chip-P4) | TGTGTTTAGTGTTGCTGGGT |
| MdAMY1-R(Chip-P4) | GCGAAAGATGGAGTGAAGGT |
| MdAMY1-F(Chip-P5) | AGCTTTCCACTAAGAGAGGA |
| MdAMY1-R(Chip-P5) | ACCCAGCAACACTAAACACA |
| MdAMY1-F(Chip-P6) | CCACGAAATTCACACCCGA |
| MdAMY1-R(Chip-P6) | GACTCCTATTCCTCTCTTAGTGG |
| MdAMY1-F(Chip-P7) | TGCATGAAAGATGTGGAGCT |
| MdAMY1-R(Chip-P7) | GGCTGCCCTTGATCTTCATT |
| MdAMY1-F(Chip-P8) | AGTGCTTTTGACTGCGTTTG |
| MdAMY1-R(Chip-P8) | GCACTTTTGTTACAGAGACAGT |
| MdAMY1-F(Chip-P9) | TGCAGAGAGAGAGAGAGAGAA |
| MdAMY1-R(Chip-P9) | GGGTGCGATGTTTATCTGATAGA |
| MdAMY1-F(EMSA) | GGGTTGGATATTTGATACAAATGGGCAGAAATAAATTGGG |
| MdAMY1-R(EMSA) | CCCAATTTATTTCTGCCCATTTGTATCAAATATCCAACCC |
| MdAMY1-Mut-F(EMSA) | GGGTTGGATATTTGATAAAAAAAGGCAGAAATAAATTGGG |
| MdAMY1-Mut-R(EMSA) | CCCAATTTATTTCTGCCTTTTTTTATCAAATATCCAACCC |
